# *Desulfatiglans-*related bacteria associated with conductive mineral particles in marine subsurface sediments

**DOI:** 10.1128/mbio.00838-26

**Published:** 2026-06-15

**Authors:** Jan V. Henkel, Hans Røy, Bo Barker Jørgensen, Amelia-Elena Rotaru, Danijel Jovicic, Ian P. G. Marshall, Chenjing Jiang, Per Halkjær Nielsen, Caitlin Margaret Singleton, Helge W. Arz, Sascha Plewe, Kasper Urup Kjeldsen

**Affiliations:** 1 Section for Microbiology, Department of Biology, Aarhus University, Aarhus, Denmark; 2 Leibniz-Institute for Baltic Sea Research Warnemünde, Rostock, Germany; 3 Department of Biology, University of Southern Denmark 6174, Odense, Denmark; 4 Center for Microbial Communities, Department of Chemistry and Bioscience, Aalborg University, Aalborg, Denmark; University of Hawaii at Manoa, Honolulu, Hawaii, USA

**Keywords:** acetate, methanogenesis, extracellular-electron-transfer, conductive minerals, microbial communities, microbial ecology, sediment, sulfate-methane-transition, desulfatiglans

## Abstract

Acetate is a key intermediate in anaerobic mineralization of organic matter in marine sediments. Recent observations suggest that acetate is oxidized syntrophically in the methanic zone of marine sediments, and that electrically conductive mineral particles could provide niches for electroactive microbial communities that perform this process. We combined radiotracer measurements, a novel procedure for ferromagnetic mineral particle extraction, and metagenomic analyses to examine this process in Baltic Sea sediments. Our results confirm that acetate is oxidized syntrophically across and below the sulfate–methane transition zones of the sediments, where the transfer of reducing equivalents from acetate oxidation to CO_2_ fuels methanogenesis. Ferromagnetic particles consistently occurred throughout the geochemical zones and mainly consisted of the electrically conductive minerals magnetite and pyrite-greigite. The microbial communities associated with ferromagnetic particles were dominated by members phylogenetically affiliated with the bacterial genus *Desulfatiglans*. Known *Desulfatiglans* species are dissimilatory sulfate reducers; however, metagenome-assembled genomes indicate that *Desulfatiglandales* populations associated with ferromagnetic particles lack genetic potential to respire sulfate. Instead, they may grow by acetate oxidation coupled with extracellular electron transfer, consistent with a conductive mineral-associated lifestyle. We hypothesize that *Desulfatiglans* relatives are acetate-oxidizing partners in a syntrophic process facilitated by interspecies electron transfer via conductive particles. We identified cytochrome-rich ANME-1 archaea as the predominant methane-cycling microorganisms associated with ferromagnetic particles; however, their potential role as methanogenic syntrophic partners remains uncertain. Overall, our study reveals that distinct microbial communities are associated with ferromagnetic particles and shows conductive minerals as a niche for electroactive microorganisms in marine sediments.

**IMPORTANCE:**

Acetate is a central intermediate in the anaerobic breakdown of organic matter. In Baltic Sea sediments at and below the sulfate–methane transition zone, we observed acetate oxidation to carbon dioxide at rates similar to methane formation from carbon dioxide reduction, a pattern indicative of syntrophic acetate oxidation. Previous enrichment studies suggest that electrically conductive mineral surfaces can facilitate this process. Motivated by this observation, we extracted ferromagnetic conductive particles from sediments and compared particle-attached microbial communities with bulk sediment. Particle-attached communities were distinct and enriched in the bacterial genus *Desulfatiglans*. Their genomes lacked genes for sulfate respiration, yet encoded traits consistent with acetate oxidation and extracellular electron transfer. Our findings suggest conductive minerals as distinct microbial niches and highlight *Desulfatiglans*-related bacteria as a potential key organism in particle-associated acetate oxidation.

## INTRODUCTION

Organic carbon mineralization in aquatic sediments proceeds via a depth-dependent successional utilization of electron acceptors, following their availability and energy yield (1). Sulfate reduction in nearshore marine sediments accounts for the major part of anaerobic organic carbon mineralization, while methanogenesis is a primary terminal mineralization process only in sulfate-depleted subsurface sediments (2, 3). Both processes are controlled by the hydrolysis and fermentation of complex organic matter, the rate of which decreases with depth and age in the sediment (4). Fermentation directly fuels the catabolism of sulfate reducers and methanogens by supplying substrates such as hydrogen and short-chain fatty acids (5).

Acetate is a fermentation product and key substrate for both sulfate reduction and methanogenesis in marine sediments (4, 6). Conventionally, acetate is oxidized completely to CO_2_ by sulfate-reducing microorganisms in the sulfatic zone and disproportionated to methane and CO_2_ by acetoclastic methanogens in the methanic zone of the sediments. However, stable isotope- and radioisotope-labeling experiments (4, 7–9) revealed that acetate is primarily oxidized to CO_2_ also in the sulfate–methane transition and methanic zones of marine sediments, while methanogenesis in these zones proceeds via CO_2_ reduction at rates similar to that of complete acetate oxidation. It was therefore hypothesized that reducing equivalents from the oxidation of acetate to carbon dioxide are used to syntrophically reduce carbon dioxide to methane (4, 10), leading to a conversion of acetate to carbon dioxide and methane that, without isotope labeling data, appears similar to acetoclastic methanogenesis. Methanogenesis via such syntrophic acetate oxidation (SAO) by acetate-oxidizing bacteria and hydrogenotrophic methanogenic partners is known from co-culture studies (11–13). Yet, the identity of such partners in the methanic zone of marine sediments and the nature of their interaction are unresolved. By enrichment culture experiments, Rotaru et al. (10, 14) showed that electrically conductive particles enable CO_2_-reductive methanogenesis in brackish and freshwater sediments by facilitating electron transfer between a bacterial acetate oxidizer and a syntrophic methanogenic partner (conductive particle-mediated interspecies electron transfer [CIET]) (15). This observation is important because marine sediments are generally rich in electrically conductive iron oxide and iron-sulfur mineral particles (16), such as hematite, magnetite, and pyrite (17–19), that are formed and transformed by physical, chemical, and biological processes, including weathering, precipitation, dissolution–reprecipitation, and oxidation–reduction reactions (19, 20). We therefore hypothesized that CIET may be of importance in the observed syntrophic acetate oxidation in marine sediments. Conductive particle-mediated interspecies electron transfer likely does not require the specialized conductive structures necessary for direct interspecies electron transfer (DIET) (10, 21) and may thus facilitate extracellular electron transfer between diverse, spatially separated syntrophic partners.

We developed a method to extract electrically conductive ferromagnetic mineral particles (FMPs) from sediment samples using neodymium magnets. We applied this procedure on sediment samples collected at three sites in the Baltic Sea, two of which are located in the Bornholm Basin and one in Aarhus Bay. We geochemically characterized the sampled sediments and measured rates of acetate oxidation to DIC, acetoclastic methanogenesis, and CO_2_-reductive methanogenesis in the sulfatic, methanogenic, and sulfate–methane transition (SMT) zones of the sediments using radiolabeled substrates. We characterized the chemical composition and abundance of FMP by automated scanning electron microscopy (SEM) and energy dispersive X-ray spectroscopy (EDX) particle analysis. We then used 16S rRNA and functional marker gene amplicon sequencing and qPCR to determine the composition and abundance of FMP-associated microbial communities and compared this to the bulk sediment microbial communities. Finally, the metabolic potential of the predominant FMP-associated community members was inferred by genome-resolved metagenomics.

## MATERIAL AND METHODS

A detailed methods section is available online as a Supplemental text.

### Sediment sampling

Intact sediment cores were collected by Rumohr Lot coring at stations BB01 (55°22.922N, 15°27.675E) and BB03 (55°28.119N, 15°28.647E) in the Bornholm Basin in June 2022 and by gravity coring at station M5 (56°06.20N, 10°27.47E) in Aarhus Bay in March 2022.

### Methane concentration and carbon cycling rates

All samples were collected on deck and preserved immediately upon core retrieval. Sediment samples for determination of porewater methane concentrations were collected using cut-off plastic syringes (4). Porewater for sulfate concentration measurements was collected by Rhizon samplers (4). Methane and sulfate concentrations were measured by gas and ion chromatography, respectively. Porosity data were obtained from earlier cruises (4, 7).

Rates of methanogenesis from dissolved inorganic carbon or acetate and rates of acetate oxidation to CO_2_ were measured by ^14^C radiotracer incubation experiments as described previously (4). For station M5, data for radiotracer-based determination of rates of anaerobic acetate and methane oxidation and methane production were taken from earlier cruises (7). The new and earlier data sets were depth-aligned based on porewater methane and sulfate concentration profiles.

### Extraction of ferromagnetic particles (FMPs)

FMPs were extracted from sediment by slurrying 200–250 cm³ samples in equal amounts of DNA- and RNA-stabilizing salt solution (22) in 750 mL plastic bottles with a neodymium block magnet attached to the outside. Slurries were placed at 4°C for 48 h on a rotational shaker. The FMPs collected by the magnet were washed by repeated suspension in fresh DNA- and RNA-stabilizing salt solution (Fig. S1).

### Automated SEM-EDX analysis of FMPs

A subset of FMP samples from each station was used to characterize the elemental composition and identity of extracted minerals (Table 2). Extracted minerals were collected on polycarbonate filters, sputter-coated with carbon, and analyzed in a scanning electron microscope (Zeiss Merlin compact) equipped with an EDX detector (X-Max 80, Oxford Instruments).

### Classification of extracted particles based on elemental composition

The mineralogy of individual particles was inferred from their elemental composition, based on cut-off values from an in-house database and verified against standard mineral samples (23). The classification scheme contains common heavy minerals with the following compositional boundary conditions (in weight %): magnetite [Fe_3_O_4_]: Si (0–5%), Ti (0–5%), and Fe (50–80%); Ti-magnetite [Fe(Fe,Ti)_2_O_3_]: Ti (5–20%) and Fe (50–70%); ilmenite [FeTiO_3_]: Ti (20–45%) and Fe (10–50%); pyrite/greigite [FeS_2_/Fe_3_O_4_]: S (12–50%) and Fe (20–60%); rutile/Ti-oxide (TiO_2_): Ti (45–60%) and Fe (0–10%); quartz [SiO_2_]: Si (35–65%); K-feldspar: Al (5–11%), Si (19–32%), K (6-14%), and Fe (0–4%); and Na-feldspar (albite): Na (5–10%), Al (6–13%), and Si (20–25%).

### DNA extraction and 16S rRNA gene PCR amplicon sequencing

Bulk sediment samples for DNA extraction were collected onboard the ship immediately upon core retrieval in 5 mL cut-off plastic syringes, which were packed in sterile Whirl-Pak plastic bags and stored at –20°C. DNA was extracted from the bulk sediment samples with DNeasy PowerLyzer PowerSoil Kit (Qiagen). For DNA extraction from FMP samples, the samples were first transferred to bead-beating tubes of the G2 DNA/RNA Enhancer Kit (Ampliqon). Bead beating was adjusted to two cycles of 15 s at 4.0 m s^−1^ and one cycle of 20 s at 5.0 m s^−1^. The subsequent steps followed the protocol of the DNeasy PowerLyzer PowerSoil Kit (Qiagen).

16S rRNA gene sequence libraries were prepared with the primer pair Univ-515F-Y and Univ-926R targeting both bacterial and archaeal 16S rRNA genes (24). Library preparation and sequencing were performed as described previously (25). Libraries were processed using cutadapt (26) and DADA2 version 1.28.0 (27). Merged forward and reverse sequences were classified against the Silva SSU Ref NR 99 v. 138 database (28) and further analyzed using the phyloseq (v1.44.0) (29) and vegan (v2.6-4) packages (30). Plots were generated in R using the ggplot2 package (v3.4.4) (31). Methyl coenzyme M reductase (*mcrA*) gene sequence libraries were constructed with the primer pair Mlas_F and McrA-rev (32, 33) and sequenced and analyzed as described and in the detailed Supplementary material and methods.

### qPCR analysis

Bacterial and archaeal 16S rRNA gene copies in DNA extracts were quantified by SYBR-Green-based qPCR, as described previously (34).

### Metagenomic sequencing, assembly, and binning

Metagenomic DNA sequence libraries were constructed from DNA extracted from sediment samples at 41, 43, and 63 cm sediment depth at station BB03.

The DNA extracts were barcoded using the Native Barcoding Kit (SQK-NBD114.24, Oxford Nanopore Technologies, Oxford, UK) according to the manufacturer’s instructions, pooled, and sequenced on a PromethION 24 sequencer (Oxford Nanopore Technologies, Oxford, UK).

Reads were processed using an automated long-read metagenomics workflow mmlong2 v0.0.4 (https://github.com/Serka-M/mmlong2) to generate metagenome-assembled genomes (MAGs). Completeness and contamination of MAGs were assessed using CheckM2 v1.0.0 (35). Genome taxonomy was assigned using GTDB-Tk v2.1.0 (36) and the Kaiju v1.9.2 database (37).

### Genome annotation

Gene calling and general functional annotation of inferred protein sequences were performed with Bakta v.1.6.1 (38) or Prokka 1.14.5 for archaeal MAGs (39). Known protein complexes involved in respiratory electron transfer were identified based on the Bakta annotation, as well as HMM models (Pfam (v35.0 [40], NCBIfam v12.0, including TIGRFAMs within the InterProScan pipeline [41]), and KEGG Orthology (42) searches. In addition, we used BLASTp v2.13.0+ (43) to search for protein homologs using Uniprot (44)-annotated proteins from known sulfate-reducing bacteria as queries, with an e-score cut-off of <e^−12^. Prediction of subcellular localization of protein-coding sequences was performed with Phobius v 1.01 (45) (integrated in the interproscan pipeline) and DeepTMHMM v.1.0.24 (46). The specific HMM models and proteins used as queries are specified in the SI Methods.

## RESULTS & DISCUSSION

We hypothesized that ferromagnetic particles (FMPs) in marine sediments provide niches for electroactive microbial communities that perform extracellular electron exchange (EET) and facilitate conductive particle-mediated interspecies electron transfer (CIET) between syntrophic acetate-oxidizing bacteria and methanogens. To test this hypothesis, we analyzed the identity and depth distribution of FMPs and their associated microbial communities across biogeochemical zones in Baltic Sea sediments.

### Acetate is oxidized syntrophically in the methanic zone

**Fig 1:**
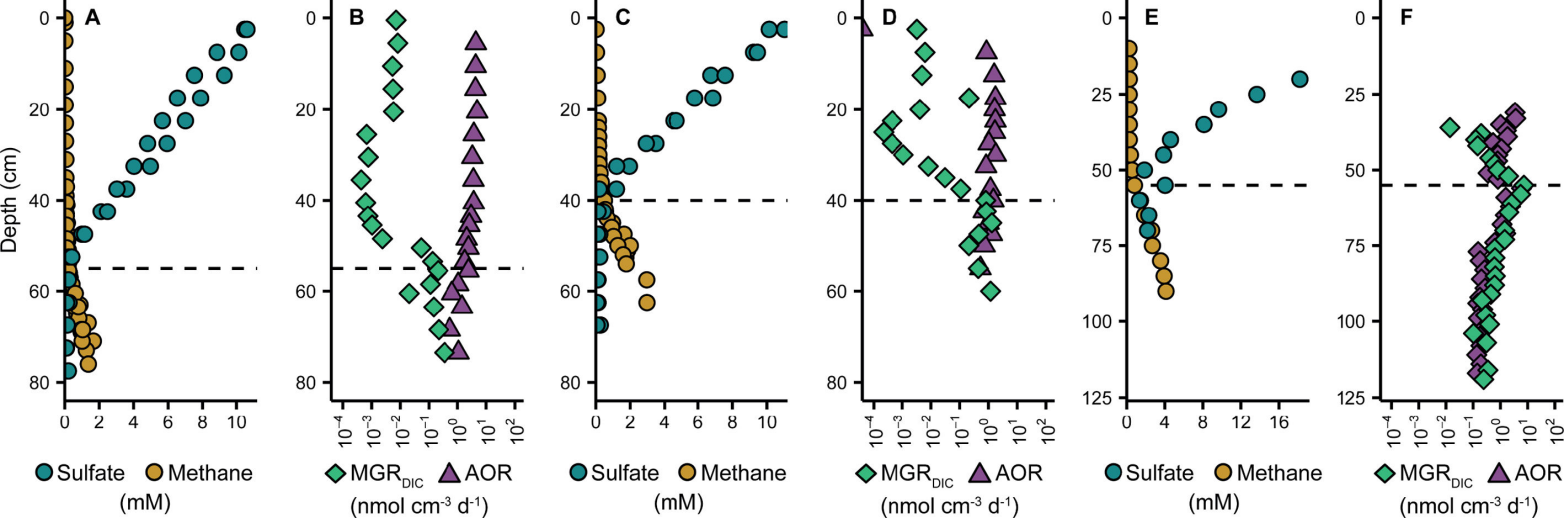
Sediment depth-resolved geochemical zonation and rates of anaerobic acetate oxidation (AOR) and methanogenesis from inorganic carbon (MGR_DIC_) at sampling stations BB01 (**A and B**) and BB03 (**C and D**) in the Bornholm Basin and station M5 (**E and F**) in Aarhus Bay. The depth of the sulfate–methane transition, where concentrations of sulfate and methane were equimolar, is indicated by a horizontal dashed line.

We collected sediment cores from three different sampling sites in the Baltic Sea: two stations in the Bornholm Basin (BB01 and BB03) at 96 m water depth and one shallower station in Aarhus Bay (M5) at 28 m water depth. Sulfate concentrations decreased steeply with sediment depth, with the sulfate–methane transition (SMT)—defined as the depth with equimolar concentrations of sulfate and methane—located between 40 and 55 cm depth (Fig. 1).

Consistently, for the three sampling stations, the rate of anaerobic acetate oxidation to CO_2_ dropped gradually about tenfold with depth down through the sulfatic and methanic zones from 1 to 10 nmol acetate cm^−3^ d^−1^ (Fig. 1). In contrast, the rate of acetoclastic methanogenesis remained low across all sampled sediment depths, with rates of 10^−4^ to 10^−3^ nmol cm^−3^ d^−1^, 100–1,000-fold lower than rates of acetate oxidation (Fig. S2), showing that acetate was primarily oxidized completely to CO_2_ in the studied sediments, and that acetoclastic methanogenesis was negligible. The CO_2_-reductive methanogenesis displayed a more distinct pattern. In the sulfatic zone, the activity was low, but toward the SMT, the CO_2_-reductive methanogenesis rate increased by two to three orders of magnitude, aligning with the rate of anaerobic acetate oxidation in the methanic zone (Fig. 1). The observed stoichiometry between acetate oxidation and CO_2_ reduction to methane in the SMT and methanic zones indicates that CO_2_-reductive methanogenesis may be fueled by reducing equivalents from acetate oxidation (7). This suggests that acetate is catabolized syntrophically via acetate-oxidizing and methanogenic microbial partners by a CIET-based mechanism (10, 14) involving FMPs present in the sediments.

### FMP speciation and distribution

**Fig 2:**
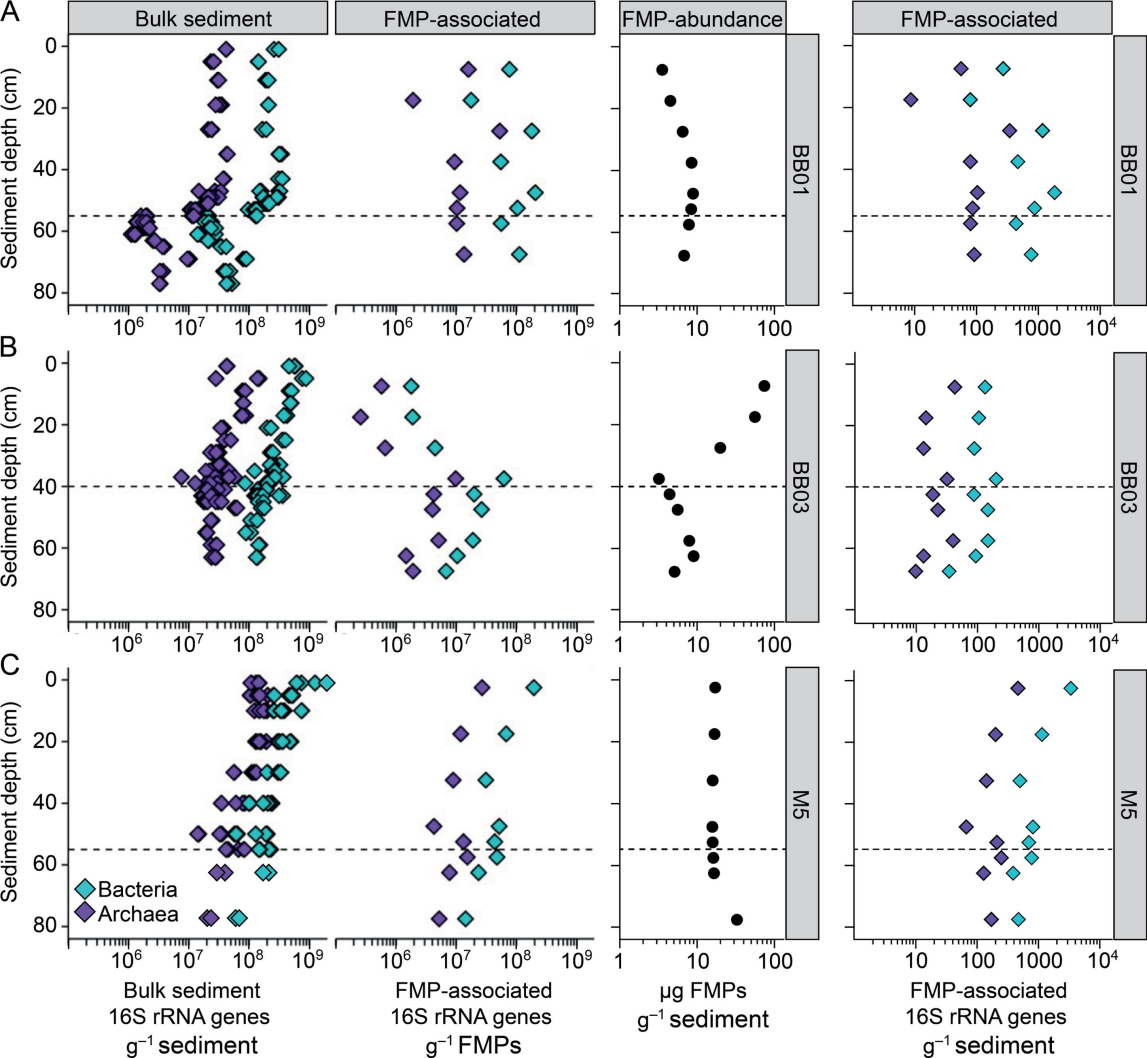
qPCR-based quantification of bacterial (turquoise color) and archaeal (purple color) 16S rRNA gene abundance in DNA extracts from bulk sediments or extracted ferromagnetic particle (FMP) samples from sampling station BB01 and BB03 in the Bornholm Basin (**A and B**) and station M5 in Aarhus Bay (**C**). The depth of the sulfate–methane transition zone in the sediments is indicated by dashed lines. The gene abundance is shown per weight of sediment and per weight of FMP, respectively. Bulk sediment samples and samples for FMP extraction were taken from parallel cores, and the former also contain FMPs.

We developed a magnetic FMP extraction procedure (Fig. S1) with which we extracted ~3,100 µg FMPs per gram of sediment depending on depth and sampling site (Fig. 2). The weight abundance of FMPs remained constant with sediment depths at station BB01 and M5 but decreased to a minimum at the SMT at station BB03 (Fig. 2). The FMP distributions are controlled by both deposition history and precipitation-dissolution by biogeochemical processes (47), and the high FMP abundance in the surface sediment of station BB03 compared to station BB01 possibly reflects the higher sedimentation rate at station BB03 (48).

It is important to note that our FMP extraction procedure likely underestimated the actual FMP abundance in the sediments because (i) not all FMPs can be captured by the magnetic extraction, (ii) FMPs can be lost during the thorough washing procedure (Fig. S1), and (iii) small FMPs cannot be retained on membrane filters with 0.2 µm pore size (filtration only done for subsequent SEM-EDX analysis). This filtration was not effective for sub-micrometer magnetite particles that are, e.g., intracellular remnants of magnetotactic bacteria (18). Furthermore, while most FMPs are electrically conductive (Table 1), they are only a subset of particles with conductive properties, many of which may not be magnetic. Therefore, our data do not fully quantify the significance of conductive mineral particles in the sediments.

**TABLE 1:** Electrical resistivity (inverse conductivity) and magnetic susceptibility of abundant mineral classes found in marine sediments^*a*^

Mineral and chemical formula	Electrical resistivity *R* (Ω M)	Mass susceptibility *k*_g_ (10^−8^ kg^−1^ m^3^)	Volume susceptibility *k* (10^−6^)	Ref.
Clay minerals/silicates				
Illite K_0.65_Al_2_[Al_0.65_Si_3.35_O_10_](OH)_2_•nH_2_O		15	410	D
Smectite (Na,Ca)_0.33_(Al,Mg)_2_Si_4_O_10_(OH)_2_•nH_2_O		2.7-5		D
Chlorite (Mg,Fe)_5_Al(Si_3_Al)O_10_(OH)_8_	1.6 × 10^9^		70-1550	TH
Quartz SiO_2_	2 × 10^14^	−0.58	−12.4	O, BP
K-feldspar				
Orthoclase KAlSi_3_O_8_	1.2 × 10^12^	−0.58	−12.5	O, BP
Carbonates				
Calcite CaCO_3_	9 × 10^13^	−0.48	−13	O, BP
Dolomite CaMg(CO_3_)_2_	4.3 × 10^13^	1.1	−38	D, O, TH
Iron oxides				
Hematite Fe_2_O_3_	10^2^	10–760	(0.05–4) × 10^4^	T, O
Magnetite Fe_3_O_4_	10^−4^	(0.2–11) × 10^4^	(1–5.7) × 10^6^	T, O
Ilmenite FeTiO_3_	(0.5–1) × 10^4^	46–8 × 10^4^	(0.002–3.8) × 10^6^	T
Titanomagnetite Fe_3-x_Ti_x_O_4_		(0.25–1.2) × 10^4^	(1.3–6.2) × 10^5^	H
Titanomaghemite Fe_2-x_Ti_x_O_3_		5.7 × 10^4^	2.8 × 10^6^	H
Iron-sulfides				
Pyrrhotite (magnetic pyrite) FeS_1+x_		(0.001–3) × 10^4^	(0.00046–1.4) × 10^6^	H
Pyrite FeS_2_	1 × 10^−3^	1–100		O, H

^*a*^ Reference key: D, Dearing (49); TH, Tarling and Hrouda (50); O, Olhoeft (51); BP, Bleil and Petersen (52); T, Telford et al. (53); H, Hunt et al. (54).

The abundance of extracted FMPs ranged from a few hundred to several thousand particles per cm^3^ sediment, with estimated surface areas of up to 10^5^ µm^2^ per cm^3^ of sediment (Fig. S3). We analyzed their elemental composition and inferred the mineralogy of more than 5,000 individual FMPs by SEM-EDX, with an average of 460 FMPs per sampled sediment depth (Table 2). It is important to note that mineral mixtures, clumping, and impurities result in a fraction of unclassified particles (Table 2) that were typically rich in iron. The observed FMP composition was highly consistent across samples from each of the three sampling stations. The FMPs from the two stations in the Bornholm Basin were predominantly composed of magnetite (Fe_3_O_4_), representing up to 70% of the analyzed FMPs (Table 2; Fig. S3). The FMPs from the surficial sediment of station BB03 were dominated by iron- and sulfur-rich particles, probably pyrite and greigite, which were also the predominant type of FMPs detected across sediment depths at the Aarhus Bay station (Table 2). The titanium minerals titanomagnetite and ilmenite represented a lower fraction of FMPs across all three sampling stations. All these iron mineral types are commonly observed in marine sediments (20, 55–57) and are considered to be electrically conductive. The specific electrical resistivity (the inverse of electrical conductivity) of mineral particles present in marine sediments ranges over orders of magnitude, from 10^14^ Ω m for inert quartz (SiO_2_) to 10^−4^ Ω m for magnetite (Fe_3_O_4_) and 10^−3^ Ω m for pyrite (FeS_2_) (58). The electrical conductivity of these particles generally correlates positively with their iron content and magnetic properties (Table 1). We therefore conclude that the FMPs may function as electrically conductive particles in the investigated sediments.

**TABLE 2:** Compositional classification (in percent) of magnetically extracted particles (ferromagnetic particles [FMPs]) from sediment cores from Bornholm Basin stations BB01 and BB03 and Aarhus Bay station M5^*a*^

Station	Depth interval (cm)	Sample size *N*	Magnetite Fe_3_O_4_	Ti-Magnetite Fe(Fe,Ti)_2_O_4_	Ilmenite FeTiO_3_	Pyrite/greigite FeS_2_/Fe_3_S_4_	Bulk sediment leftovers			Other classes and unclassified by Fe content (Fe content in weight %)							
							Na-feldspar	K-feldspar	Quartz SiO_2_	Fe >45	Fe 40–45	Fe 30–40	Fe 20-–30	Fe 10–20	Fe 5–10	Fe 1–5	Fe 0–1
BB01	10–15	464	52	10	5	0.4	1	0	2	11	3	5	2	2	2	2	1
	40–45	465	69	6	1	0	3	2	4	4	1	1	1	3	3	2	1
	70–75	457	72	5	2	0.4	0.2	0.4	2	5	4	2	2	2	2	0.2	1
BB03	10–15	449	13	0	1	37	1	0.4	10	2	1	4	2	11	12	6	1
	30–35	466	40	7	10	0.4	2	2	4	12	5	2	3	4	3	2	4
	60–65	420	47	2	15	0.5	2	0.2	3	2	1	1	4	6	3	3	10
	70–75	446	38	5	13	0.2	2	1	16	8	3	3	1	3	0.4	0.2	9
M5	10–15	453	6	2	6	22	7	7	21	1	0.2	0.4	1	5	12	7	3
	40–45	479	9	2	14	25	3	4	15	2	1	1	1	7	9	3	6
	70–75	477	3	0	8	39	4	5	13	0.2	0.2	1	3	8	9	4	2
	85–90	482	2	0.2	7	49	3	4	11	0.4	0	0.2	2	8	7	4	2

^*a*^ Sample size (*N*) indicates the number of particles that have been analyzed with SEM-EDX and do not reflect particle abundance within samples. Feldspar and quartz are considered dominant mineral types found in Baltic Sea sediments (e.g., 59, 60) and are indicative of incomplete purification of FMPs from the bulk sediment matrix (bulk sediment leftovers).

We used qPCR of 16S rRNA genes to quantify the absolute abundance of bacterial and archaeal communities in bulk sediment and the FMP-associated bacterial and archaeal communities. In bulk sediments, the bacterial and archaeal abundances decreased with depth by an order of magnitude within the uppermost 60 to 80 cm (Fig. 2). The FMP-associated bacterial and archaeal abundances at station BB01 did not steadily decrease with sediment depth compared to bulk sediment samples, while at station BB03, the abundances increased within the studied sediment depth, peaking at the SMT. In contrast, at Aarhus Bay station M5, the abundances of FMP-associated bacteria and archaea decreased by an order of magnitude with sediment depth, mirroring the trend observed in the bulk sediment samples (Fig. 2). Considering the weight abundance of FMPs, a gram of sediment contained 100–1,000 FMP-associated 16S rRNA genes, and the FMP-associated microbial abundance thus appeared orders of magnitude outnumbered by the bulk sediment microbial abundance (Fig. 2). A minor abundance of FMP-associated microorganisms could be expected when considering the estimated surface area of extracted FMPs, which was 10,000–100,000 µm^2^ per cm^3^ sediment (Fig. S3). If the cells covered a projected area of 0.5–1 µm^2^ (61), the FMPs could only host 0.1% of cells in the bulk sediment, even if their surface was completely populated by a monolayer of cells. However, this calculation must be taken with caution, since the extraction procedure underestimates the in-situ FMP abundance (and the associated microbial community) by an unknown factor as described earlier. Furthermore, nucleic acid extraction from highly enriched metal-bearing minerals can result in reduced yields (62). These observations suggest that our qPCR data likely underrepresents the true abundance of microorganisms associated with electrically conductive mineral particles in these marine sediments.

### *Desulfatiglans*-affiliated bacteria associate with FMPs

**Fig 3:**
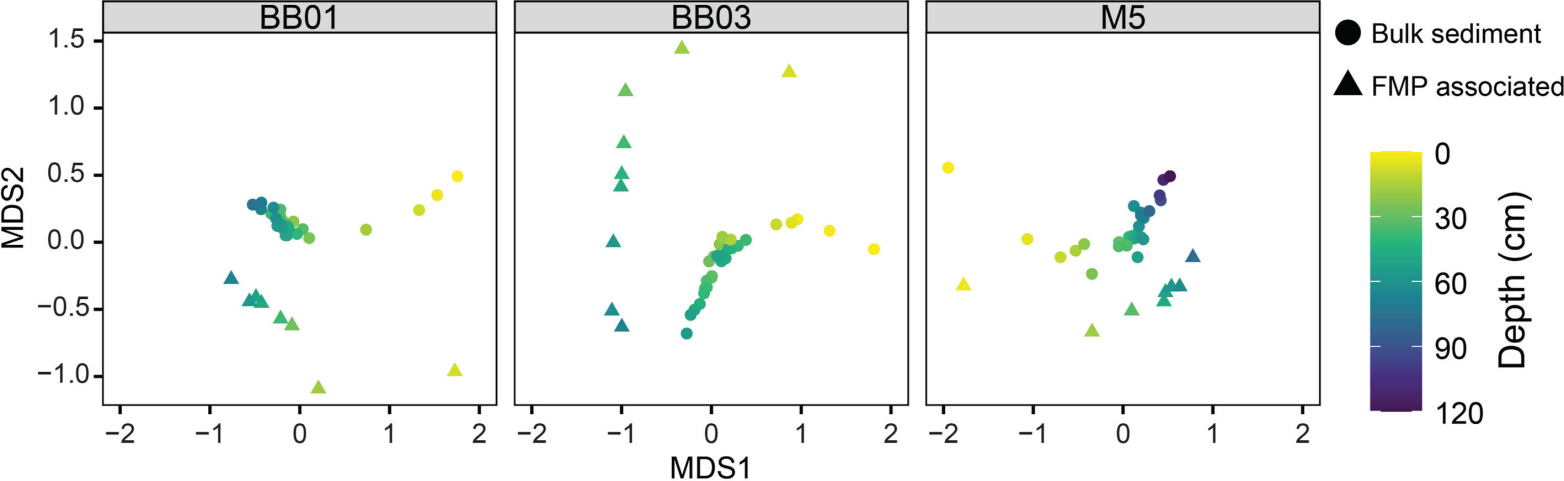
Non-metric multidimensional scaling-based ordination analysis of microbial community composition of bulk sediment and ferromagnetic particle (FMP) samples from sampling stations BB01 and BB03 in the Bornholm Basin and station M5 in Aarhus Bay. The ordinations were based on analyses of the ASV-level composition of 16S rRNA gene amplicon sequence libraries using Bray-Curtis dissimilarity. Sediment depth is indicated by a color gradient.

We characterized the composition of the microbial communities associated with FMPs by 16S rRNA gene amplicon sequencing. We initially validated the reproducibility of this procedure on FMP samples extracted from eight independent sediment cores collected in Aarhus Harbor (see Supplementary Discussion and Fig. S4). In the Bornholm Basin and Aarhus Bay sediments, the species (16S rRNA gene ASV) composition of the bulk sediment changed with sediment depth, with the most pronounced changes occurring across the uppermost 10–20 cm (Fig. 3), which include the steepest changes in sediment geochemistry (63). The species composition of the FMP-associated communities was clearly different from that of the bulk sediment at all analyzed depths, yet it still mirrored the depth-dependent change in bulk species composition (Fig. 3). This suggests that the FMP-associated communities assembled in a dynamic exchange with the bulk microbial community across all analyzed depths of the sediments.

**Fig 4:**
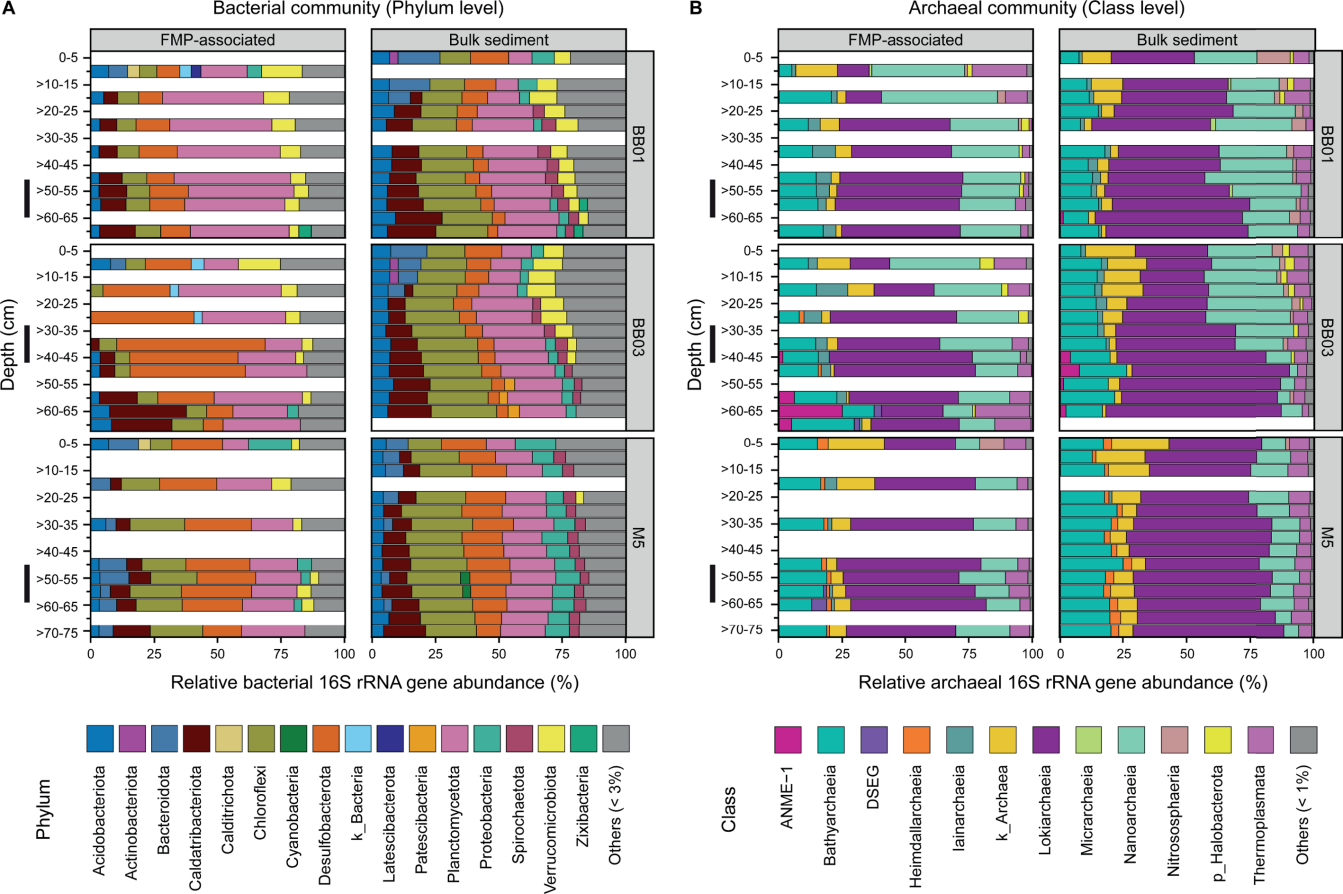
Phylum-level bacterial (**A**) and class-level archaeal (**B**) community structure based on universal 16S rRNA gene amplicon sequencing of ferromagnetic particle (FMP) and from bulk sediment samples from sampling stations BB01 and BB03 in the Bornholm Basin and station M5 in Aarhus Bay. Bacterial phyla that were below 3% relative abundance and archaeal phyla that were below 1% were summed as “Others.”

As is typical for organic matter-rich marine sediments (e.g., 34, 64), the bacterial communities were dominated by the phyla *Chloroflexi*, *Desulfobacterota*, *Planctomycetota*, and *Caldatribacteriota,* while the archaeal communities were dominated by classes *Lokiarchaeia*, *Nanoarchaeia*, *Bathyarchaeia*, and *Thermoplasmata* (Fig. 4). In bulk sediment communities, the phylum *Desulfobacterota* was mainly represented by the uncultivated genus SEEP-SRB1 and the genus *Desulfatiglans* (Fig. S5), which are common microbial community members of organic matter-rich marine sediments (63, 65, 66). While the relative abundance of SEEP-SRB1 decreased with increasing sediment depth, the relative abundance of *Desulfatiglans* increased, peaking at the SMTZ at all stations (Fig. S5). Interestingly, the FMP-associated communities were strongly dominated by *Desulfatiglans*, irrespective of the sampling station (Fig. S5), with relative abundances four to five times higher than in the bulk sediment. The FMP-associated depth distribution of this genus otherwise mirrored that of the bulk sediment, with a peak relative abundance at the SMTZ, most notably at station BB03, where up to 50% of the reads in the FMP-derived 16S rRNA gene amplicon sequence libraries were taxonomically classified as *Desulfatiglans*.

**Fig 5:**
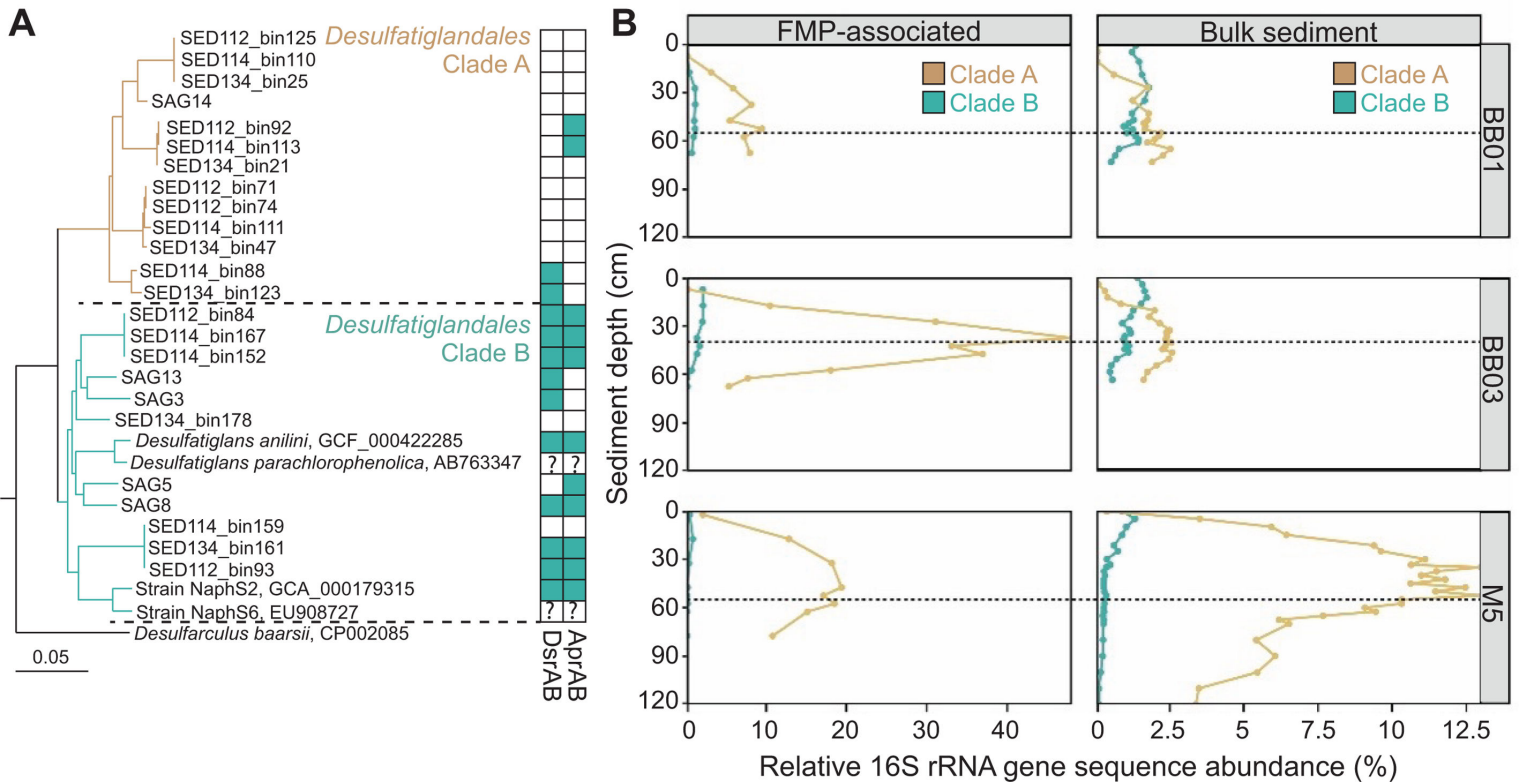
Phylogeny and sediment depth distribution of *Desulfatiglans*-affiliated lineages at sampling stations BB01 and BB03 in the Bornholm Basin and station M5 in Aarhus Bay. (**A**) 16S rRNA gene sequence-based phylogeny of metagenome-assembled genomes (MAGs [name beginning with “SED”]) and single-cell amplified genomes (SAGs) affiliated with the order *Desulfatiglandales*. Clade A represents taxa lacking the genes *dsrAB* and *aprAB,* encoding key enzymes in dissimilatory sulfate reduction pathways, while clade B represents MAGs/SAGs which harbor these genes (as indicated by filled green squares), as well as isolated and characterized dissimilatory sulfate reducers. Note that genome sequences are not available for the sulfate-reducing isolates *D. parachlorophenolica* and strain NaphS6, but their sulfate-reducing capabilities have been shown experimentally (52, 70) (indicated by empty, but colored, squares). The phylogenetic tree was estimated by distance matrix analysis using the pos_var_ssuref:bacteria filter of the Silva SSU Ref v138 ARB database (53) for selecting alignment positions for the analysis. The scale bar shows 5% estimated sequence divergence. The MAGs were derived from metagenomic sequencing of sediment samples from station BB03, and SAGs were derived from a previous study of sediment samples from Aarhus Bay station M5 (54). (**B**) Sediment depth distribution of reads from 16S rRNA gene amplicon sequence libraries from ferromagnetic particle (FMP) or bulk sediment samples that map to either *Desulfatiglandales* clade A or clade B. The depth of the sulfate–methane transition zone in the sediments is indicated by dashed lines. See Fig. S9 for details of the sequence read mapping and for a version of the phylogenetic tree including its full outgroup.

Cultivated and characterized members of the genus *Desulfatiglans* conserve energy by dissimilatory sulfate reduction (67, 68). However, a single-cell genome (SAG)-based study indicated that some environmental *Desulfatiglans*-affiliated populations may lack the genetic capacity for this catabolic process (69). We used genome-resolved metagenomics to explore the metabolic potential of the populations represented by the *Desulfatiglans*-affiliated 16S rRNA gene sequences observed in our amplicon sequence libraries. Specifically, we focused on identifying possible roles in acetate oxidation, energy conservation, and extracellular electron transfer. By Nanopore sequencing of DNA extracted from three different depths at station BB03, we obtained 29 MAGs phylogenetically affiliated with *Desulfatiglans* (Table S1, Fig. S6). According to both genome and 16S rRNA gene sequence-based phylogenies, the MAGs formed a monophyletic group with the two named species of the genus *Desulfatiglans* (68) and the related strains NaphS2 (67) and NaphS6 (70) (Fig. 5A; Fig. S6). While some MAGs formed a clade, which we named “clade B,” with the known cultivated members and relatives of the genus *Desulfatiglans,* the rest formed a sister clade, which we named “clade A” (Fig. 5A; Fig. S6). The clade A MAGs are taxonomically classified within the GTDB family B25-G16 of the order *Desulfatiglandales*, while the clade B MAGs classify within the family *Desulfatiglandaceae* of the same order (Table S1, Fig. S9). In agreement, *Desulfatiglandales* clade A MAGs share less than 75% average nucleotide identity (ANI) with any clade B MAGs in pairwise comparisons, clearly indicating that clade A and B MAGs represent distinct genera and families (71) (Table S2).

Among the 29 *Desulfatiglans*-affiliated MAGs, 18 harbored a 16S rRNA gene sequence (Fig. 5A; Fig. S6), which allowed us to match their phylogenetic identity with that of the ASVs in our 16S rRNA amplicon sequence libraries (see Fig. S6 for details). Consistent for all three sampling stations, *Desulfatiglandales* clade B ASVs were of higher relative abundance than clade A ASVs in the surface sediment, but decreased in relative abundance with sediment depth (Fig. 5B). The depth distribution of *Desulfatiglandales* clade B ASVs is thus like that of SEEP-SRB1 members (Fig. S5) and correlates with the decreasing sulfate reduction rate with sediment depth (4, 7). In contrast, *Desulfatiglandales* clade A ASVs increased in relative abundance with sediment depth, peaking at the SMT, and outnumbered clade B ASVs already in the upper part of the sulfatic zones (Fig. 5B). This pattern was highly pronounced in the FMP-associated communities, where clade A ASVs reached relative abundances of up to 50% (Fig. 5B). To test whether this depth-related difference between clade A and clade B was unique to our data set, we mapped our MAG-derived 16S rRNA gene sequences against 16S rRNA gene amplicon sequence data from other studies of microbial communities across the sulfatic and methanic zones of marine sediments (64, 72). We observed opposing depth distribution of clade A and B members: clade B declined with sediment depth, while clade A increased (Fig. S7). Single *Desulfatiglandales* clade A ASV-level sublineages drove the high *Desulfatiglans* abundance in both the Bornholm basin and the Aarhus Bay sediments, and in each case, the sublineages were represented by several near-identical MAGs originating from different sediment depths (Fig. S8). In contrast to other parts of the FMP-associated community, which changed dynamically with sediment depth (Fig. 3), these *Desulfatiglandales* clade A sublineages clearly seem to persist across depth and geochemical zones of the sediment.

### Respiratory metabolism of *Desulfatiglans*-affiliated MAGs

Metabolic reconstruction of the 29 *Desulfatiglans*-affiliated MAGs revealed a distinct metabolic divergence within the *Desulfatiglandales*. Most clade A MAGs lacked genes encoding key enzymes and electron transfer complexes essential for dissimilatory sulfate reduction (73), including adenosine 5′-phosphosulfate reductase (AprAB), dissimilatory sulfite reductase (DsrAB), and the two conserved transmembrane electron-transferring complexes DsrMKJOP and quinone-interacting membrane-bound oxidoreductase complex QmoABC (74). A few MAGs affiliated with clade A contained genes encoding one of the subunits of the AprAB or DsrAB enzymes (Fig. S9). Consequently, we extended our analysis of the presence of these marker genes to include published GTDB species-representative MAGs that are phylogenetically most closely related to the MAGs examined in this study. The results confirmed the general absence and scattered distribution of the DsrAB and AprAB in the clade A MAGs (Fig. S9). In contrast, clade B MAGs contained the genes encoding DsrAB and AprAB and associated complexes (73). These results indicate that clade A members are unlikely to perform dissimilatory sulfate reduction, in agreement with earlier predictions by Jochum et al. (69). This raises the critical question of how clade A members conserve energy.

**Fig 6:**
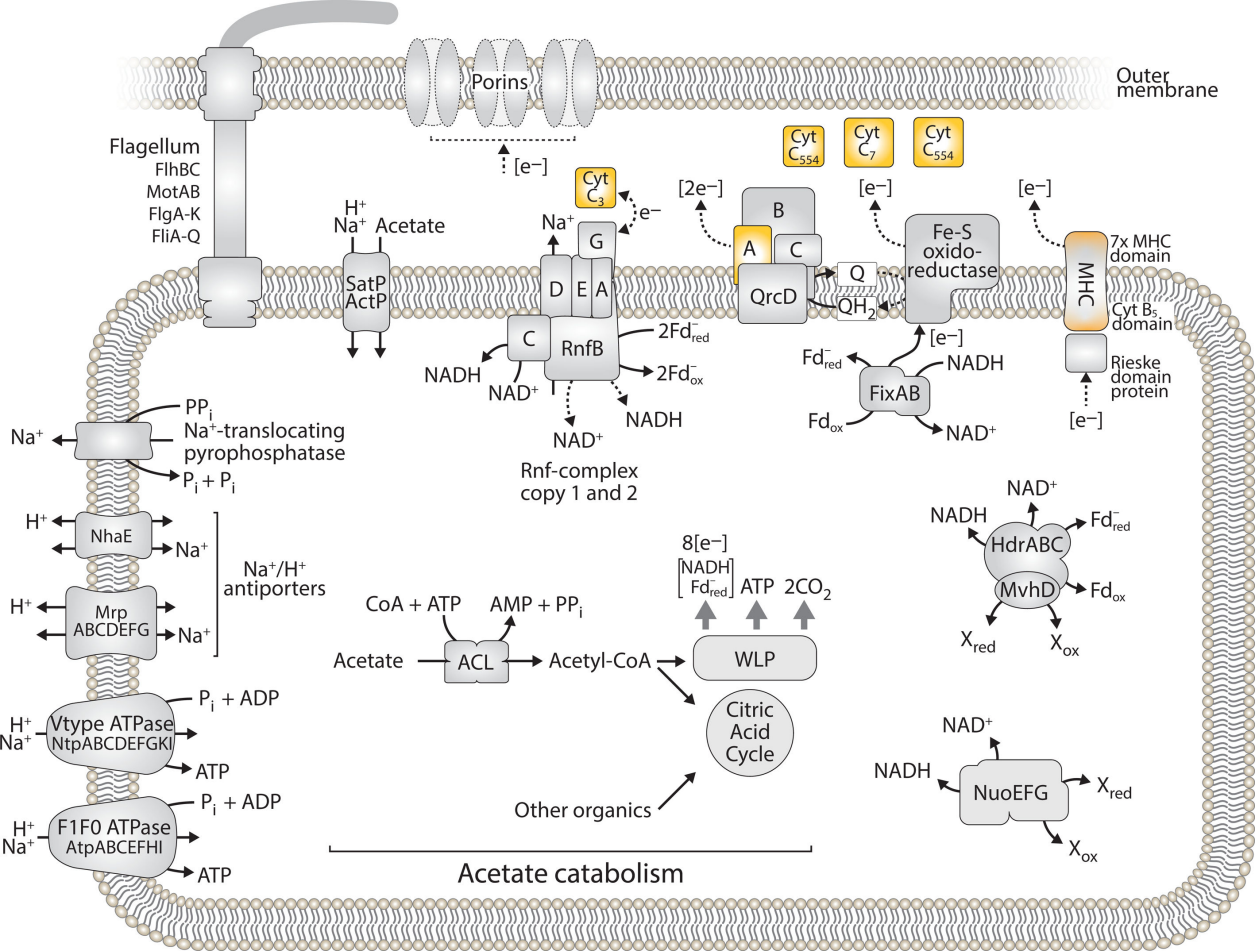
Genome-inferred metabolic model of *Desulfatiglandales* clade A members. See main text and SI data for details. ACL, AMP-forming acetyl-CoA synthetase3. WLP, Wood-Ljungdahl Pathway. Multiheme cytochromes are marked in orange color. Dotted lines indicate hypothetical processes. See the SI material for a discussion of the HdrABC-MvhD and NuoEFG complexes.

Despite these differences in sulfate reduction capabilities, both clades appear capable of oxidizing organic carbon. Isolated strains of *Desulfatiglans* (e.g., *D. anilini* and NaphS2) can grow on acetate, and their genomes encode the full Wood-Ljungdahl pathway (69), which is used for acetate oxidation in *D. anilini* (75). The clade A and clade B MAGs harbor the genes encoding this pathway, starting with the AMP-forming acetyl-CoA synthetase, which converts acetate, CoA, and ATP into acetyl-CoA, AMP, and pyrophosphate (76) (Fig. 6). This enzyme system has a higher affinity for acetate compared to the conventional acetate kinase/phosphotransacetylase system (77) and operates irreversibly due to the hydrolysis of pyrophosphate. The latter may be catalyzed by a membrane-bound sodium-extruding pyrophosphatase (Fig. 6), which would furthermore help limit the energy cost of activating acetate (78). Given that both clades encode this pathway, we hypothesize that both clades can oxidize acetate, generating NADH and reduced ferredoxin. Energy is potentially conserved by the Rnf complex, which is found consistently in clade A and B members. This complex translocates Na^+^ across the cytoplasmic membrane by the reduction of NAD^+^ to NADH with reduced ferredoxin as electron donor (79) (Fig. 6). An electron-bifurcating ETF:quinone oxidoreductase complex, known from *Desulfococcus multivorans* (80), may couple the exergonic transfer of electrons from two cytoplasmic NADH to the membrane (mena)quinone pool (81) with the endergonic reduction of ferredoxin (82) via a membrane-bound iron-sulfur oxidoreductase encoded next to *etfAB* in the MAGs (Fig. 6). This complex likely increases the amount of reduced ferredoxin that can be linked to energy conservation via the Rnf-complex (81).

The *Desulfatiglandales* clade A and B MAGs encode a F1F0 ATPase and multisubunit H^+^/Na^+^ antiporter that may enable ATP production from a sodium or proton motive force (Fig. 6). The *Desulfatiglandales* clade B MAGs, as well as the genomes of strain NaphS2 and *D. anilini*, contain two to four *atpE* copies that encode the membrane rotor-forming C subunit of the ATPase complex (83). Genomes of anaerobic Rnf-complex-dependent bacteria regularly contain multiple *atpE* copies, which may allow them to modulate ion binding by the ATPase (84). Most *Desulfatiglandales* clade A MAGs only harbor a single *atpE* copy. However, *Desulfatiglandales* clade A MAGs uniquely encode a V-type ATPase and a NhaE family H^+^/Na^+^ antiporter (Fig. 6). F1F0- and V-ATPases may both generate or dissipate either a sodium or proton motive force (83). We were unable to resolve the H^+^ or Na^+^ specificity of the ATPases encoded in the *Desulfatiglandales* MAGs based on alignment of their C-subunits (85); however, the observed distinct ATPase and H^+^/Na^+^ antiporter composition in clade A suggests a unique energy metabolism of the populations represented by these MAGs compared to clade B members.

### Putative energy metabolism of FMP-associated *Desulfatiglandales*

Based on analyses of a limited number of single-cell amplified genomes, and unaware of their remarkable FMP association, we previously proposed that *Desulfatiglandales* clade A members populating marine sediments conserve energy via acetogenesis (69). The acetyl-CoA pathway can operate in both directions: oxidative for acetate oxidation and reductive for acetogenesis and CO_2_ assimilation (86). Consequently, the direction of the pathway cannot be determined from genomic evidence alone (87). Furthermore, some acetogens are electroactive and can accept electrons from cathodes or syntrophic partners (88). We therefore cannot rule out the possibility that *Desulfatiglandales* clade A members conserve energy via acetogenesis rather than via syntrophic acetate oxidation (SAO). However, given the importance of acetate oxidation in the sediments (Fig. 1) and the predominance of *Desulfatiglandales* (Fig. 5), we consider it more likely that this group is involved in acetate oxidation than in acetogenesis.

*Desulfatiglandales* clade A members were highly abundant (Fig. 5), with a genetic potential for acetate oxidation and extracellular electron transfer but lacking the potential to perform dissimilatory sulfate reduction (Fig. 6). We therefore speculate that *Desulfatiglandales* clade A members associate with conductive mineral particles, such as FMPs, and use them as electron sinks for a respiratory-type catabolism, and that such an extracellular electron transfer may facilitate a CIET-based syntrophic oxidation of acetate. Electrons transferred to the membrane-bound quinone pool may be passed to periplasmic c-type cytochromes via a quinone-reducing QrcABCD complex (89). The clade A MAGs consistently encode four such multiheme cytochromes, including a C_7_-domain cytochrome and a C_554_-domain cytochrome that are absent in clade B MAGs (Fig. 6; Table S1). Additionally, both clade A and clade B MAGs encode a C_3_-domain cytochrome c protein within the same operon as the RNF complex, which may facilitate electron exchange with this complex, similar to the mechanism proposed for SRB (90) and *Methanosarcina acetivorans* (91). Clade A MAGs also encode a membrane-spanning protein that contains both a periplasmic cytochrome C_7_ domain and a cytoplasmic cytochrome b5 domain. The gene encoding this protein is located adjacent to a Rieske protein-encoding gene. These proteins may interact to shuttle electrons between the cytoplasm and periplasm, helping to maintain redox balance along with the cytoplasmic HdrABC–MvhD and the NuoEFG complexes, which are also encoded by clade A MAGs (Fig. 6).

Electron transfer from the periplasm across the outer membrane typically involves multiheme cytochromes and outer membrane-spanning, beta barrel-forming porin conduits (92). We searched the *Desulfatiglandales* MAGs for homologs of proteins known to form these conduits in *Shewanella*, *Geobacter,* and *Rhodopseudomonas,* including MtrABC and OmcABC (93), ExtABC (94), and PioAB (95), as well as OetABI and OmcLK, which have both been identified in sulfate-reducing partners of methanotrophic archaea (92). We were unable to detect such homologs based on amino acid sequence identity. Compared to clade B MAGs, clade A MAGs contain several unique hypothetical porin-encoding genes that could function in EET (Fig. 6; Table S1). None of them were located adjacent to multiheme cytochrome-encoding genes in the MAGs, and their possible role in extracellular electron transfer is thus putative.

The FMP-associated communities from the Bornholm Basin sediment were dominated by ASVs representative of clade A MAG bins SED112_bin125, SED114_bin110, and SED134_bin25. Compared to other clade A and clade B MAGs, the three MAGs contained all eight magnetosome membrane (*mamABEIKMPQ*) (96) formation genes that are considered essential to form intracellular magnetite (Fe_3_O_4_) or greigite (Fe_3_S_4_) inclusions in magnetotactic bacteria (see Supplementary Discussion for details). Magnetotactic bacteria are known to live at oxic/anoxic interfaces, where magnetotaxis enables them to position within oxygen gradients and navigate relative to these gradients (97, 98). That cannot be the case for the putative magnetotactic sublineage of *Desulfatiglandales* clade A that populates anoxic subsurface sediments without such gradients. It is tempting to speculate that magnetotaxis could be used to find magnetic particles with conductive properties, such as FMPs, by sensing gradients in their magnetic field (99), as previously hypothesized (100). We cannot exclude the possibility that intracellular magnetic minerals contribute to the accumulation of magnetotactic cells in the FMP fraction due to our sampling method, where FMPs were collected using strong magnets, but we consider this scenario unlikely for the following reasons. Magnetotactic bacteria align to an external magnetic field for directed flagellar movement, but they are not passively attracted by magnets (101). Thus, dead cells of magnetotactic bacteria were reported to align passively within a magnetic field, but cells with crystalline magnetite and greigite magnetosomes were not pulled by magnet forces (100–102). Further research is needed to elucidate the ecological niche of *Desulfatiglandales* clade A and their potential magnetotactic behavior.

### Methanogens were rarely detected

While rate measurements show methane production from ^14^CO_2_ (MGR_DIC_, Fig. 1), methanogens were rare in our 16S rRNA gene sequence libraries from bulk sediment and FMP samples (Fig. 4; Fig. S10). Only two known methanogenic lineages, *Methanofastidiosales* and *Methanomassiliicoccales,* were detected and mainly occurred in the sulfatic or in the SMT and methanic zones, respectively (Fig. S10). Members of these methanogen orders likely conserve energy by hydrogen-dependent reduction of methyl groups (49) and may therefore not be involved in the CO_2_ dependent methanogenesis characteristic of the studied stations and depths (Fig. 1). Comparing the low relative abundances of these methanogenic lineages in bulk sediment or the FMP-associated communities, there is little evidence of their enrichment in the FMP community. Furthermore, these methanogens are outnumbered by orders of magnitude by members of the *Desulfatiglans* lineage in the FMP communities (Fig. 5; Fig. S10). Our 16S rRNA gene sequence libraries indicate that ANME-1 archaea were scarce in the sulfate zone but became abundant at the SMT, exceeding the relative abundance of known methanogens by one to two orders of magnitude, both at this depth and in the underlying methanic zone (Fig. S10). We further evaluated this pattern by *mcrA* amplicon sequencing of bulk sediment and FMP samples from the SMT and methanic zone at station BB03. The resulting *mcrA* libraries were dominated by three ANME-1–affiliated sequence types (Fig. S11), each of which could be mapped to the *mcrA* genes encoded by three closely related ANME-1 MAGs (Fig. S11 and S12). Notably, each MAG contained two closely related *mcrA* copies; however, one copy in each genome is truncated by ~300 nt at the 5′ end and is located adjacent to a recombinase gene. This arrangement suggests that the truncated *mcrA* gene product is likely nonfunctional (Fig. S10).

ANME-1 archaea have been suggested to carry out methanogenesis by reversing their methane-oxidation pathway (7, 50, 65, 66); however, to date, there is no experimental evidence demonstrating methane formation by ANME-1 cells to support this hypothesis. The three ANME-1 MAGs encode a complete methanogenesis pathway that could potentially operate in reverse to support anaerobic methane oxidation (Fig. S11). Although ANME-1 archaea are thought to interact with sulfate-reducing bacterial partners to facilitate sulfate-dependent methane oxidation, the mechanisms that allow syntrophic electron exchange remain poorly understood (51). Notably, the ANME-1 MAGs do not encode the large S-layer-spanning multiheme cytochromes associated with direct interspecies electron transfer (DIET) in ANME-2 archaea (51). Instead, they encode several smaller extracytoplasmic multiheme cytochromes that perhaps could serve an analogous role in mediating direct electron transfer with a syntrophic partner (Fig. S11). We speculate that ANME-1 perform methanogenesis in the SMT and methanic zones of the sediment in a CIET-dependent partnership with acetate-oxidizing microorganisms. However, this remains a working hypothesis that currently lacks experimental validation.

Finally, it should be noted that our FMP and DNA extraction procedures may have failed to detect certain methanogenic lineages. For example, the extensive washing steps used during FMP extraction (Fig. S1) could differentially affect FMP-associated cells depending on their attachment mechanisms, with some cells potentially being more sensitive and prone to lysis during processing.

The observation of a non-sulfate-reducing *Desulfatiglandales* clade A was discussed in relation to their acetate-oxidizing potential and its connection to canonical methanogens via electrically conductive particles. However, since this is speculative, we do also want to mention the possibility that clade A could accept electrons from their environment and act as homoacetogens, or that the electrons from acetate oxidation could be transferred to sulfate reducers, although we consider this as unlikely.

### Conclusion

Our radiotracer rate measurements showed that acetate was oxidized completely to CO_2_ in the SMT and methanic zones of Baltic Sea marine subsurface sediments with stoichiometric reduction of CO_2_ to CH_4_, indicating syntrophic acetate oxidation as the main pathway for methanogenesis. We hypothesize that this process is driven by a syntrophic partnership involving CIET. Supporting this idea, we identified a lineage of *Desulfatiglans*-related bacteria consistently associated with conductive particles in the SMT and methanic zones of the sediments. Unlike their closely related sulfate-reducing relatives, this lineage lacks the genomic capacity for dissimilatory sulfate reduction yet retains the potential for acetate oxidation. We propose that these organisms may transfer electrons derived from acetate oxidation to conductive mineral particles. However, further investigation is needed to clarify the ecological role of this abundant lineage in marine subsurface sediments. The low abundance of canonical CO_2_-reducing methanogens in zones of active CO_2_-reductive methanogenesis once again raises the question of whether ANME-1 may play a dual role, performing not only anaerobic oxidation of methane but also methanogenesis. Overall, our study offers a framework for studying microbial interactions with electrically conductive minerals in marine sediments.

## Data Availability

The 16S rRNA gene PCR amplicon sequence library data and raw Nanopore sequence data were deposited in NCBI SRA database under BioProject accession numbers PRJNA1176456 and PRJNA1126655, respectively. The MAGs are stored with the identifiers SAMN57186025 to SAMN57186056 (> 30 in total).

## SUPPLEMENTAL MATERIAL

Supplemental text mbio.00838-26-s0001.docx Detailed material and methods; supplemental discussion.

Supplemental material mbio.00838-26-s0002.docx Supplemental tables and figures.

Supplemental data set mbio.00838-26-s0003.xlsx Gene identifiers and accession numbers in reported MAGs.

ASM does not own the copyrights to Supplemental Material that may be linked to, or accessed through, an article. The authors have granted ASM a non-exclusive, world-wide license to publish the Supplemental Material files. Please contact the corresponding author directly for reuse.
