# Supplementary material for: Desulfatiglans-related bacteria associated with conductive mineral particles in marine subsurface sediments
Source: mBio. 2026 Jun 15;17(7):e00838-26. doi: 10.1128/mbio.00838-26 (PMC13343851; doi:10.1128/mbio.00838-26)
Supplement: Supplemental text — Detailed material and methods; supplemental discussion. [file mbio.00838-26-s0001.docx]

#### Supplementary Information

**Title**

*Desulfatiglans-*related bacteria associated with conductive mineral particles in marine subsurface sediments

Running title (max 54 characters incl. spaces): *Desulfatiglans* spp. associate with conductive minerals

**Authors**

Jan V. Henkel^1,4,#^, Hans Røy^1^, Bo Barker Jørgensen^1^, Amelia-Elena Rotaru^2^, Danijel Jovicic^2^, Ian P. G. Marshall^1^, Chenjing Jiang^3^**,** Per Halkjær Nielsen ^3^, Caitlin Margaret Singleton ^3^, Helge W. Arz ^4^, Sascha Plewe ^4^, Kasper Urup Kjeldsen^1,#^

**Affiliation**

1 Section for Microbiology, Department of Biology, Aarhus University, Aarhus, Denmark

2 Department of Biology, University of Southern Denmark, Odense, Denmark

3 Center for Microbial Communities, Department of Chemistry and Bioscience, Aalborg University, Aalborg, Denmark

4 Leibniz-Institute for Baltic Sea Research Warnemünde, Rostock, Germany

^#^**Corresponding authors**

[jan.henkel@io-warnemuende.de](mailto:jan.henkel@io-warnemuende.de)

kasperuk@bio.au.dk

**This document includes**

Supplementary materials and methods and supplementary discussion.

Supplementary Table S1 and Figures S1-S10.

Supplementary References

**Supplementary Information Methods**

**Porewater methane and sulfate**

Sampling for porewater to determine the concentration of methane depended on the core type. In Rumohr (1) cores, 2.5 mL cut-off plastic syringes were laterally inserted into the sediment core that was step-wise extruded into a predrilled ring for syringe insertion made from a section of the core liner. Gravity cores were cut in 1-m sections and immediately sampled for methane by pushing a 2.5 mL cut-off syringe through drilled holes at selected sampling depths. Samples for methane were immediately transferred to 20 mL glass vials pre-filled with 2.5 mL saturated NaCl and closed with butyl rubber stoppers, crimp capped, shaken, and stored upside down at –20 °C. Methane concentrations were measured by injecting 200 µL of the gas phase into a gas chromatograph equipped with a 0.9 m packed silica gel column (3.1 mm inner diameter) and a flame ionization detector (GC-FID; SRI 310C; SRI Instruments). After methane sampling, the gravity core liner was opened lengthwise with a vibrating saw and the top half of the core liner was removed to allow sampling in the inner part of the core. Porewater for sulfate analysis was sampled using Rhizon samplers as previously described (2): Holes were drilled into intact Rumohr lot cores and Rhizon samplers were inserted into the sediment. In gravity cores, Rhizon samplers were inserted through cling film covering the exposed sediment to reduce exposure to air. A 1.5 mL subsample of extracted porewater was stripped of hydrogen sulfide by bubbling with water-saturated CO_2_ gas and stored at 4 °C until analysis. Sulfate pore water concentrations were determined by ion chromatography as described earlier (3).

**^14^C radiotracer incubations**

Rate measurements with ^14^C-labelled substrates were performed as described earlier (2). In short, aediment samples for determining rates of methanogenesis from dissolved inorganic carbon or acetate, and rates of acetate oxidation to CO_2_, were taken laterally from sediment cores similar to methane samples using syringe-like rubber stoppered glass tubes equipped with rubber-stoppered plungers. The use of glass tubes enabled effective retention of methane during incubation. We added 10 µL ^14^C labelled substrate (aiming for the addition of 10 kBq or 100 kBq with 2-^14^C-acetate or ^14^C-DIC, respectively) with glass syringes to the middle of the subcore and incubated the rubber stoppered glass tubes at in-situ temperature (9 °C) for 10 days in sealed gas-tight plastic bags together with an Oxoid AnaeroGen (Thermo Scientific) oxygen scrubber. Direct contact of the sachet with the sample tubes was avoided to prevent the initial heat production of the AnaeroGen during O_2_ removal from heating the samples. Incubations were stopped by transferring enclosed sediment with the surrounding glass tube into 20 mL crimp neck glass test tubes, prefilled with 10 mL 2.5% NaOH, immediately sealing with a butyl rubber stopper, crimp capped, shaken, and stored upside down at –20 °C. To liberate the incubated sediment from the inner incubation tube and mix it with the hydroxide solution inside the closed test tubes, the samples were thawed and refrozen to break up the sediment cohesion. Radioactivity in the methane, DIC and acetate pools was then separated (2). For killed controls two sediment subcores, one taken directly above and one directly below the subcore samples incubated for rate measurements, were injected with the radiolabelled tracer and immediately terminated as described above. Sediment depth-resolved porewater concentrations of acetate and DIC for rate calculations were taken from (2) for Bornholm Basin stations BB01 and BB03 and from (4) for Aarhus Bay station M5.

**Extraction of ferromagnetic particles (FMPs)**

For extraction of FMPs, Rumohr Lot sediment cores from station BB01 and BB03 were cut into 5 cm sections, which were vacuum-sealed in gas-impermeable plastic bags (Mitsubishi Gas Chemicals, High Gas Barrier Bag) with a chamber vacuum sealer (Caso VacuChef 70) and then stored within a secondary gas-tight bag containing Oxoid AnaeroGen at 4 °C for 7 days until processed further. The gravity core from station M5 was processed immediately in 5 cm sections. The sediment sections were transferred to sterile 750 mL plastic bottles containing an equivalent volume of sterile filtered DNA and RNA stabilizing salt solution (5) and slurried by vigorous shaking.

For harvesting ferromagnetic particles (FMPs) a neodymium block magnet (dimensions: 18 x 15 x 10 mm; approximate holding force: 96.1 N) was affixed to the exterior of the plastic bottles using electrical tape. This setup was then placed on a rotational shaker at 140 RPM at 4 °C for 48 hours. Subsequently, the sediment slurry was discarded and the FMPs that adhered to the bottle side facing the magnet were resuspended with fresh DNA and RNA stabilizing solution and carefully transferred into a 2 mL microcentrifuge tube with a sterile pipette. The extracted FMPs were washed three times by resuspension in 2 mL fresh DNA and RNA stabilizing solution to dislodge loosely attached particles, then magnetically attracted to the tube sides while removing the supernatant. The purified FMPs, with the residual DNA and RNA stabilizing solution adhering to the particles, were subsequently stored at -80 °C pending DNA extraction (Fig. S1).

**Automated SEM-EDX analysis of FMPs**

The analyzed FMPs were first washed three times in 2 mL sterile Milli-Q water to remove any residual salts from the DNA and RNA stabilizing solution. The FMPs were then dried overnight at 60 °C and weighed. To disaggregate the dried particles, we resuspended them in 2 mL sterile Milli-Q water in a microcentrifuge tube and subjected them to ultrasonic treatment for 3 minutes in a water bath ultrasonicator. Depending on FMP concentration between 0.01 and 0.5 mL of the 2 mL suspension was filtered onto a 0.2 µm polycarbonate filter with 20 mm diameter immediately after sonification, aiming to have a sparse but even coverage on the filter, barely visible by the naked eye. The filters were dried at 60 °C, vacuum sputter-coated with carbon, glued to aluminum studs, and loaded into a scanning electron microscope (Zeiss Merlin compact) equipped with an EDX unit (X-Max 80 detector, Oxford instruments). Data recording and analysis were done with the Oxford Aztech 3.3 software and automated particle identification and analysis were done with Oxford AZtechFeature. Individual particles were identified by contrast (electron density) against the low-density polycarbonate carrier. A maximum of 10 particles per area (150 µm x 110 µm) and a minimum of 400 per sample were mapped by EDX. The number of identified particles per scanned area per sample was used to back-calculate FMP abundance in the sediment. The surface area (*S*) of each particle was assumed to be ellipsoid and calculated according to:

$$S = 4\pi\left( \frac{\left( \frac{a\times b}{2} \right)^{1.6}+\left( \frac{a\times b}{2} \right)^{1.6}+\left( \frac{b}{2} \right)^{1.6}}{3} \right)^{\frac{1}{1.6}}$$

where *a* and *b* represent half of the measured length and width of the particles.

**Preparation of 16S rRNA and *mcrA* gene amplicon sequence libraries**

For DNA extraction from bulk sediment samples approximately 0.25 g sediment was transferred to PowerBead Tubes and mixed with 300 µL DNA extraction buffer (100 mM Tris-HCl, 100 mM Na-EDTA, 100 mM sodium phosphate, 1.5 M NaCl, 1% cetrylmethylammonium bromide, pH 8.0). The samples were then mechanically disrupted by bead beating in a FastPrep-24™ 5G Bead beating Grinder and Lysis System (MP Biomedicals) for two cycles of 40 s at 5.5 m s^-1^ and subjected to a two-step enzymatic lysis (6). In the first step 50 µL of a lysozyme, lipase, pectinase and β-glucuronidase enzyme mixture (10 µg mL^-1^ of each enzyme) was added to the homogenized sediment and incubated in a thermomixer for 30 min at 37 ^o^C. In the second step, 50 µL of a mixture of proteinase K, protease and pronase (10 µg mL^-1^ of each enzyme) was added and similarly incubated for 30 min at 37 ^o^C. Subsequently, the homogenized and digested sediment was mixed with 75 µL 20% (w/v) SDS and incubated at 65 ^o^C for 60 min in the thermomixer. Hereafter the DNA extraction and purification were performed following the protocol of the kit with a bead beating step as described above.

Attempts to extract DNA from FMP samples using the standardized procedures were initially unsuccessful. We therefore modified the protocol by omitting the enzymatic and chemical lysis steps and first transferred FMP samples to the bead beating tubes of the G2 DNA/RNA enhancer kit (Ampliqon). Bead beating was adjusted to two cycles of 15 seconds at 4.0 m s^-1^ and one cycle of 20 seconds at s 5.0 m s^-1^. The subsequent steps followed the protocol of the DNeasy PowerLyzer PowerSoil Kit (Qiagen), and DNA was finally eluted in 35 µL of the provided elution buffer.

Bacterial and archaeal 16S rRNA gene fragments were PCR amplified from the extracted DNA with the primer pair Univ-515F-Y and Univ-926R (7). The reaction mixture included 12.5 µL of KAPA HIFI HotStart ReadyMix 2X (Roche), 0.5 µL bovine serum albumin solution (10 μg μL^-1^), 0.5 µL of each primer (both at 10 pmol µL^-1^), 6 µL of dH_2_O, and 5 µL of template DNA. Thermal cycling included initial denaturation at 95°C for 3 minutes, followed by 20 (bulk sediment) or 24 (FMP samples) cycles of 95°C for 30 seconds, 50°C for 30 seconds, and 72°C for 30 seconds, with a final extension step at 72°C for 5 minutes.

PCR amplication of *mcrA* fragments was achieved with the primer pair Mlas_F and McrA-rev (8) targeting the *mcrA* of both methanogenic and methanotrophic archaea (9). The PCR reactions mixtures were the same as used for the 16S rRNA gene amplification. Thermal cycling included initial denaturation at 95°C for 3 minutes, followed by 20 (bulk sediment) or 30 (FMP samples) cycles of 95°C for 30 seconds, 53°C for 30 seconds, and 72°C for 30 seconds, with a final extension step at 72°C for 5 minutes.

The resultant PCR products were supplied with forward and reverse Illumina adapter overhang sequences in a second round of PCR in preparation for sequencing on an Illumina Miseq using a 600 cycle MiSeq v3 Reagent Kit (Illumina). The negative control PCR reactions from the first round of PCR were included in the second round and did not yield an amplification product as evaluated by agarose gel electrophoresis.

**Amplicon gene sequence library analysis**

Raw 16S rRNA gene amplicon reads from the MiSeq were initially trimmed by cutadapt (10) hereby keeping only reads with perfect match to primer sequences and trimming off the primer sequences. The trimmed reads were further processed using DADA2 version 1.28.0 (11). Forward reads were trimmed to a length of 240 nt and reverse reads to a length of 200 nt with the “filterAndTrim” function using the setting “maxN=0, maxEE=c(2,2), truncQ=2”. Error models were inferred from the trimmed reads with the “LearnErrors” function, reads were dereplicated with the “derepFastq” function and denoised into amplicon sequence variants (ASVs) with the “dada” function. The resultant read pairs were merged with the “mergePairs” function with default settings, including only merged sequences with a length between 350 and 425 nt. The sequences were screened for chimera with the “removeBimeraDenovo” function. Finally, using the “assignTaxonomy” function the 16S rRNA gene sequences were classified taxonomically against the Silva SSU Ref NR 99 v. 138 database (12). Phyloseq objects were generated using the phyloseq package (v1.44.0) (13) and ordination was done with vegan (v2.6-4) (14). Grouping on selected phylogenetic levels, calculation of domain specific abundances, assigning the level “others” to groups below defined cut-off values etc. was done with the dplyr package (v1.1.3) (15) on melted phyloseq objects generated with the psmelt function within the phyloseq package

The *mcrA* sequence libraries were processed similarly to the 16S rRNA gene libraries using Cutadapt and DADA2 to trim, denoise, and merge forward and reverse reads, followed by clustering into chimera-screened amplicon sequence variants (ASVs). The resulting ASVs were translated in all six reading frames, and the amino acid sequences were queried against the 2021 version of the COG database (16) using RPS-BLAST (17) with an E-value cutoff of 0.001. Amino acid sequences that lacked internal stop codons and matched COG4058 (McrA, methyl coenzyme M reductase, alpha subunit) were retained for further analysis. These sequences were aligned using MUSCLE (18) with default settings. The alignment was imported into the ARB software package (19) for manual curation and to calculate an uncorrected similarity matrix. Based on this matrix, the amino acid sequences were clustered into operational taxonomic units (OTUs) at a 99% identity cutoff using Mothur (20). Phylogenetic classification of the OTUs was performed by adding OTU-representative sequences to the amino acid alignment of a public *mcrA* database (mcrA4All; (21)) and constructing maximum likelihood phylogenetic tree using RaxML version 7.2.8 (22).

**Metagenomic sequencing, assembly and binning**

For construction of metagenomic DNA sequence libraries, DNA was extracted from 10 replicate 0.25 g sediment samples from each of 3 different sediment depths (41, 43 and 63 cmbsf) spanning the SMTZ at station BB03. The DNA extraction was performed as described above for the 16S rRNA gene sequence library analysis using the DNeasy PowerLyzer PowerSoil Kit with the G2 DNA/RNA enhancer kit and the preprogrammed bead beating setting ‘marine sediment’ in the FastPrep-24™ instrument. The replicate DNA extracts were subsequently pooled by loading on the MB Spin column of the DNeasy PowerLyzer PowerSoil Kit and eluted in two steps with 40 and 20 µL elution solution supplied with the kit. Subsequently, the extracts were purified and size selected on a BluePippin instrument (Sage Science) with a BLF7510 0.75% agarose gel cassette collecting a fragment range from 3000 to 15000 bp. The resultant size selected extracts had a DNA concentration of 19-21 ng DNA µL^-1^ with an average fragment size of 7000-10000 bp. Barcoded (Native Barcoding kit (SQK-NBD114.24, Oxford Nanopore Technologies, Oxford, UK) DNA extracts were pooled in equimolar amounts and sequenced on a PromethION 24 sequencer using the R10.4.1 flow cells (FLO-PRO114M, Oxford Nanopore Technologies, Oxford, UK). The flow cell ran >72 h at a translocation rate of 400 nt per second. Raw Nanopore data were basecalled with Guppy (v. 6.3.9, https://community.nanoporetech.com/downloads) in super-accurate mode with the trim barcodes function on. The sequence data yield ranged from 25 to 55.7 Gbp depending on the sample with an estimated N_50_ of 5.21 kbp. The quality of the long reads was evaluated using NanoStat (23).

Metagenome-assembled genomes (MAGs) were generated from the Nanopore sequence data with the automated long-read metagenomics workflow mmlong2 v0.0.4 (<https://github.com/Serka-M/mmlong2>) as follows. Reads were assembled with Flye v2.9.1 (24) with ‘--meta --min-overlap 0 --extra-params min_read_cov_cutoff=3’. Then the assemblies were polished via Medeka v1.7.2 (https://github.com/nanoporetech/medaka) using the subprogram medaka consensus and medaka stitch commands. Circular contigs (n=20) were separated from the polished assemblies to form a subset of circular MAGs, leaving the remaining non-circular contigs for further MAG reconstruction. Contig coverage was estimated with minimap2 v2.24 (25) and the script ‘jgi_summarize_bam_contig_depths’ from MetaBAT2 (26) and used as input for the binning process. Binning utilized multiple tools (MetaBAT2 v2.15 (26), GraphMB v0.1.5 (27), and SemiBin v1.4.0 (28)) and underwent several rounds to yield the final refined MAGs (n=669). Initially, the three binning tools were separately run on the non-circular contigs, following default settings with consistent cutoffs for minimum contig length and minimum MAG length, set at 3,000 bp and 250,000 bp, respectively. The resultant binning output was then combined and refined with the DAS Tool v1.1.3 (29) using the default setting (--score_threshold=0.5).

Average nucleotide identity (ANI) values were calculated using fastANI (30). Shown ANI and alignment fraction values represent averages of bidirectional pairwise comparisons of MAGs.

**Genome annotation**

Key enzymes for dissimilatory sulfate reduction DsrABC, AprAB and DsrMKJOP were identified with a blastp search using the sequences of *Desulfobacula toluolica* strain DSM 7476 as queries. Genes of the Rnf complex (RnfABCDEG) were identified with TIGRfam (TIGR01943-47), Pfam (PF0250, PF13375, and PF03116), and blastp search against the amino acid sequence from respective Rnf genes identified via Hidden-Markov-Model in MAG bin SED134_bin25. The quinone-interacting membrane bound oxidoreductase QmoABC complex was identified with blastp search using protein sequences from *Desulfovibrio desulfuricans* ATCC 27774 (31, 32) as queries. The NADH:quinone oxidoreductase (Nuo) complex was identified with blastp search using protein sequences from *Desulfatiglans* sp. strain NaphS2 as queries. The electron bifurcating electron transfering flavoprotein ETF:quinone (etf) oxidoreductase complex was identified with a blastp search against sequences from *Syntrophomonas wolfei* strain DSM 2245B and *Desulfococcus multivorans* (33) together with a membrane bound iron-sulfur oxidoreductase (Dmul_c0100 – from *D. multivorans*). The membrane bound QrcABCD complex (34, 35) was identified by blastp search using protein sequences from *Nitratidesulfovibrio* *vulgaris* ATCC 29579 (formerly known as *Desulfovibrio vulgaris*) as queries. Essential magnetosome membrane (mam) genes *mamA*, *B*, *E*, *I*, *K*, *M*, *P*, and *Q* (36) were identified by sequence similarity with respective genes from *Magnetospirillum gryphiswaldense* strain MSR-1 (37) and *Candidatus* Magnetoglobus multicellularis strain BW-1 (38). MamI was not reported for *Ca.* M. multicellularis strain BW-1 in (38) and was identified via NCBIfam NF040963. Specific gene loci are given in the supplementary table. The multisubunit H^+^/Na^+^ antiporter was identified by match to NCBI Conserved Domains PRK07375, PRK12507, PRK12651, PRK12668 and PRK12674. The NhaE-family H^+^/Na^+^ antiporter was identified by blastp search with the characterized protein (Q4QSA9) of *Rhodothermus marinus* as query. Citric acid cycle enzymes were identified based on the Bakta annotation. The Wood Ljungdahl pathway enzymes were identified by blastp search using the protein sequences of *Moorella thermoacetica* as queries (39), The Na^+^ translocating pyrophosphatase was identified by TiGRfam TIGR01104. The F1F0-type and V-type ATPase complexes were identified by based on the Bakta annotation and checked using the V-type subunit amino acid sequences of *Enterococcus hirae* (Q08636, Q08637, P43456, P43435, P43436, P43437, P43455, P43457, P43439) and the F1F0-type subunit amino acid sequences of *E. coli* (P0ABB0, P68699, P0AB98, P0ABA4, P0ABC0, P0ABA0, P0A6E6) as blastp queries.

To identify of extra-cytoplasmic multi-heme cytochromes and outer membrane proteins potentially involved in EETWe searched for heme binding motifs, including the canonical CX_2_CH and non-canonical variants (CX_3_CH to CX_15_CH (40–43)) in gene products with an inferred extra-cellular localization. Proteins with least 6 heme binding sites were cross-referenced with the COG database for further functional classification. We assigned protein sequences to the same cluster in case the COG number were the same and the amino acid sequence was of comparable lengths (within a 20-50 AA range – manually checked). Proteins lacking COG assignments were grouped based on inferred homology by amino acid sequence alignment. Proteins sharing >50% amino acid sequence identity across >75% of their length were grouped together. Our analysis identified 68 clusters through COG database comparison and 9 additional groups with no COG assignment, totaling 77 distinct groups.

We used DeepTMHMM (44) to identify potential beta barrel porin-forming gene products that include 14 or more and an even number of beta sheets and therefore likely embedded in the outer membrane (45). We specifically focused the analysis on gene products with a potential function in EET i.e. lacking an auto-annotation that would otherwise suggest involvement in transport of molecules across the outer membrane.

Homologous proteins were identified by clustering proteins into groups based on amino acid sequence identity using MMseqs2 (46) with settings as specified in the SI Data.

**Supplementary Information Discussion**

**Validation of FMP Extraction and Non-Random Assembly of FMP-Associated Communities**

To confirm that our FMP-associated microbial communities were not random subsets of the bulk sediment microbiome, we conducted parallel extractions of sediments from Aarhus Harbor. These independent extractions showed reproducible patterns of both community composition and the identity of predominant species consistently attached to FMPs (Fig. S4). This reproducibility strongly suggests that the observed FMP-associated communities are a distinct and non-randomly assembled subpopulation. Although the stringent washing protocols likely underestimated the total microbial abundance on FMPs, the consistent enrichment of specific taxa on FMPs across samples underscores the ecological relevance of these mineral-attached communities.

***Planctomycetota* putative family SG8-4 are also enriched on FMPs**

Within the phylum of Planctomycetota, which showed selective enrichment of FMP particles (Fig. 4), the putative family SG8-4 (Silva Taxonomy) was dominant across all sampled depths in bulk sediments with up to 10% relative abundance and up to 20% in the FMP-associated fraction. The relative abundance of this group was enrichhed no more than 2-fold in FMP-associated fraction compared to the bulk sediment samples, and increased in relative abundance with sediment depth, but didt not exhibit the defined peak at the SMT zone as observed for *Desulfatiglandales* (Fig. 5).

**Clade A *Desulfatiglandales* MAGs containing sulfate reduction pathway genes**

The clade B MAGs encoded homologs of the QmoA and QmoB subunits of the QmoABC complex which shared share 40-50% full-length amino acid sequence identity with the functionally characterized QmoAB subunits of *Desulfovibrio desulfuricans* (31, 32) (see SI table). The QmoAB-encoding genes occur in synteny with *aprAB* which is typical for sulfate-reducing bacteria (47), as well as with a gene encoding a putative QmoC subunit that however only share 25% amino acid sequence identity with the QmoC of *D. desulfuricans.* Similarly to the *D. desulfuricans* QmoC the clade B QmoC also includes 6 transmembrane helices in its C-terminal part and a cytoplasmic N-terminal part with an 4Fe-4S binding domain.

In contrast, the clade A MAGs encode no obvious QmoABC homologs. The MAGs do encode proteins which share 25% full length amino acid sequence identity with *D. desulfuricans* QmoA and B, but the genes encoding these proteins generally do not occur in synteny with each other (see SI table) and were annotated as heterodisulfide reductase and hypothetical proteins.

The clade B MAGs encoded homologs of the QmoA and QmoB subunits of the QmoABC complex which shared share 40-50% full-length amino acid sequence identity with the functionally characterized QmoAB subunits of *Desulfovibrio desulfuricans* (31, 32) (see SI table). The QmoAB-encoding genes occur

**Metabolic potential of *Desulfatiglans*-related MAGs**

The *Desulfatiglandales* clade A MAGs harbor an operon that encode HdrABC and MvhD which likely forms a cytoplasmic complex that can interconvert NADH and reduced ferredoxin (48). They furthermore encode the cytoplasmic dehydrogenase subunits NuoEFG of the Nuo complex while lacking all its membrane-associated genes (49). The NuoEFG may similarly facilitate electron exchange between NADH and cytoplasmic redox partners. Together with the ETF:quinone oxidoreductase these complexes may balance fueling a respiratory RNF complex with reduced ferredoxin and NAD^+^ (Fig. 6) (50).

Based on limited single cell genomics evidence we previously proposed that *Desulfatiglandales* clade B members may conserve energy by acetogenesis (51). Although ACS is considered a main acetate-activating enzyme forming acetyl-CoA functioning in the oxidative direction of the Wood-Ljungdahl pathway (52) it was recently shown to catalyze the reverse reaction in an acetogenesis pathway of methanogenic archaea (53). Therefore, we cannot rule out that the clade B members as an alternative to extracellular electron transport can conserve energy by acetogenesis. The *Desulfatiglandales* clade A and B MAGs furthermore encode putative phenyl phosphate carboxylases and benzoyl-CoA reductases that are involved in aromatic compound activation and degradation in *D. anilini* and its cultured relatives (54, 55). The MAGs furthermore encode putative reductive dehalogenase (RdhA)-enzymes (see SI table). As discussed previously (51), this suggest that *Desulfatiglandales* clade A and B members can catabolize aromatic compounds and possibly respire using halogenated compounds as electron acceptors.

**References:**

1. Meischner D, Rumohr J. 1974. A Light-weight, High-momentum Gravity Corer for Subaqueous Sediments.

2. Beulig F, Røy H, Glombitza C, Jørgensen BB. 2018. Control on rate and pathway of anaerobic organic carbon degradation in the seabed. Proceedings of the National Academy of Sciences 115:367–372.

3. Flury S, Røy H, Dale AW, Fossing H, Tóth Z, Spiess V, Jensen JB, Jørgensen BB. 2016. Controls on subsurface methane fluxes and shallow gas formation in Baltic Sea sediment (Aarhus Bay, Denmark). Geochim Cosmochim Acta 188:297–309.

4. Lever MA, Torti A, Eickenbusch P, Michaud AB, Å antl-Temkiv T, JÃ¸rgensen BB. 2015. A modular method for the extraction of DNA and RNA, and the separation of DNA pools from diverse environmental sample types. Front Microbiol 6.

5. Juretschko S, Timmermann G, Schmid M, Schleifer K-H, Pommerening-Röser A, Koops H-P, Wagner M. 1998. Combined Molecular and Conventional Analyses of Nitrifying Bacterium Diversity in Activated Sludge: *Nitrosococcus mobilis* and *Nitrospira* -Like Bacteria as Dominant Populations. Appl Environ Microbiol 64:3042–3051.

6. Parada AE, Needham DM, Fuhrman JA. 2016. Every base matters: assessing small subunit rRNA primers for marine microbiomes with mock communities, time series and global field samples. Environ Microbiol 18:1403–1414.

7. Steinberg LM, Regan JM. 2009. *mcrA* -Targeted Real-Time Quantitative PCR Method To Examine Methanogen Communities. Appl Environ Microbiol 75:4435–4442.

8. Deng L, Bölsterli D, Glombitza C, Jørgensen BB, Røy H, Lever MA. 2025. Drivers of methane-cycling archaeal abundances, community structure, and catabolic pathways in continental margin sediments. Front Microbiol 16.

9. Martin M. 2011. Cutadapt removes adapter sequences from high-throughput sequencing reads. EMBnet J 17:10.

10. Callahan BJ, McMurdie PJ, Rosen MJ, Han AW, Johnson AJA, Holmes SP. 2016. DADA2: High-resolution sample inference from Illumina amplicon data. Nat Methods 13:581–583.

11. Quast C, Pruesse E, Yilmaz P, Gerken J, Schweer T, Yarza P, Peplies J, Glöckner FO. 2012. The SILVA ribosomal RNA gene database project: improved data processing and web-based tools. Nucleic Acids Res 41:D590–D596.

12. McMurdie PJ, Holmes S. 2013. phyloseq: An R Package for Reproducible Interactive Analysis and Graphics of Microbiome Census Data. PLoS One 8:e61217.

13. Oksanen J, Blanchet FG, Friendly M, Kindt R, Legendre P, McGlinn D, Minchin PR, O’hara RB, Simpson GL, Solymos P, others. 2022. vegan: Community ecology package (2.6-4). See https://github. com/vegandevs/vegan.

14. Wickham H, François R, Henry L, Müller K, Vaughan D. 2023. dplyr: A Grammar of Data Manipulation.

15. Galperin MY, Wolf YI, Makarova KS, Vera Alvarez R, Landsman D, Koonin E V. 2021. COG database update: focus on microbial diversity, model organisms, and widespread pathogens. Nucleic Acids Res 49:D274–D281.

16. Camacho C, Coulouris G, Avagyan V, Ma N, Papadopoulos J, Bealer K, Madden TL. 2009. BLAST+: architecture and applications. BMC Bioinformatics 10:421.

17. Edgar RC. 2004. MUSCLE: multiple sequence alignment with high accuracy and high throughput. Nucleic Acids Res 32:1792–1797.

18. Ludwig W. 2004. ARB: a software environment for sequence data. Nucleic Acids Res 32:1363–1371.

19. Schloss PD, Westcott SL, Ryabin T, Hall JR, Hartmann M, Hollister EB, Lesniewski RA, Oakley BB, Parks DH, Robinson CJ, Sahl JW, Stres B, Thallinger GG, Van Horn DJ, Weber CF. 2009. Introducing mothur: Open-Source, Platform-Independent, Community-Supported Software for Describing and Comparing Microbial Communities. Appl Environ Microbiol 75:7537–7541.

20. Lever MA, Alperin MJ, Hinrichs K-U, Teske A. 2023. Zonation of the active methane-cycling community in deep subsurface sediments of the Peru trench. Front Microbiol 14.

21. Stamatakis A. 2014. RAxML version 8: a tool for phylogenetic analysis and post-analysis of large phylogenies. Bioinformatics 30:1312–1313.

22. De Coster W, D’Hert S, Schultz DT, Cruts M, Van Broeckhoven C. 2018. NanoPack: visualizing and processing long-read sequencing data. Bioinformatics 34:2666–2669.

23. Kolmogorov M, Bickhart DM, Behsaz B, Gurevich A, Rayko M, Shin SB, Kuhn K, Yuan J, Polevikov E, Smith TPL, Pevzner PA. 2020. metaFlye: scalable long-read metagenome assembly using repeat graphs. Nat Methods 17:1103–1110.

24. Li H. 2018. Minimap2: pairwise alignment for nucleotide sequences. Bioinformatics 34:3094–3100.

25. Kang DD, Li F, Kirton E, Thomas A, Egan R, An H, Wang Z. 2019. MetaBAT 2: an adaptive binning algorithm for robust and efficient genome reconstruction from metagenome assemblies. PeerJ 7:e7359.

26. Lamurias A, Sereika M, Albertsen M, Hose K, Nielsen TD. 2022. Metagenomic binning with assembly graph embeddings. Bioinformatics 38:4481–4487.

27. Pan S, Zhu C, Zhao X-M, Coelho LP. 2022. A deep siamese neural network improves metagenome-assembled genomes in microbiome datasets across different environments. Nat Commun 13:2326.

28. Sieber CMK, Probst AJ, Sharrar A, Thomas BC, Hess M, Tringe SG, Banfield JF. 2018. Recovery of genomes from metagenomes via a dereplication, aggregation and scoring strategy. Nat Microbiol 3:836–843.

29. Jain C, Rodriguez-R LM, Phillippy AM, Konstantinidis KT, Aluru S. 2018. High throughput ANI analysis of 90K prokaryotic genomes reveals clear species boundaries. Nat Commun 9:5114.

30. Duarte AG, Santos AA, Pereira IAC. 2016. Electron transfer between the QmoABC membrane complex and adenosine 5′-phosphosulfate reductase. Biochimica et Biophysica Acta (BBA) - Bioenergetics 1857:380–386.

31. Pires RH, Lourenço AI, Morais F, Teixeira M, Xavier A V, Saraiva LM, Pereira IAC. 2003. A novel membrane-bound respiratory complex from Desulfovibrio desulfuricans ATCC 27774. Biochimica et Biophysica Acta (BBA) - Bioenergetics 1605:67–82.

32. Dörries M, Wöhlbrand L, Kube M, Reinhardt R, Rabus R. 2016. Genome and catabolic subproteomes of the marine, nutritionally versatile, sulfate-reducing bacterium Desulfococcus multivorans DSM 2059. BMC Genomics 17:918.

33. Venceslau SS, Lino RR, Pereira IAC. 2010. The Qrc membrane complex, related to the alternative complex III, is a menaquinone reductase involved in sulfate respiration. Journal of Biological Chemistry 285:22774–22783.

34. Pereira IAC, Ramos AR, Grein F, Marques MC, da Silva SM, Venceslau SS. 2011. A Comparative Genomic Analysis of Energy Metabolism in Sulfate Reducing Bacteria and Archaea. Front Microbiol 2.

35. Liu P, Zheng Y, Zhang R, Bai J, Zhu K, Benzerara K, Menguy N, Zhao X, Roberts AP, Pan Y, Li J. 2023. Key gene networks that control magnetosome biomineralization in magnetotactic bacteria. Natl Sci Rev 10.

36. Awal RP, Lefevre CT, Schüler D. 2023. Functional expression of foreign magnetosome genes in the alphaproteobacterium *Magnetospirillum gryphiswaldense*. mBio 14.

37. Lefèvre CT, Menguy N, Abreu F, Lins U, Pósfai M, Prozorov T, Pignol D, Frankel RB, Bazylinski DA. 2011. A Cultured Greigite-Producing Magnetotactic Bacterium in a Novel Group of Sulfate-Reducing Bacteria. Science (1979) 334:1720–1723.

38. Ragsdale SW, Pierce E. 2008. Acetogenesis and the Wood–Ljungdahl pathway of CO2 fixation. Biochimica et Biophysica Acta (BBA) - Proteins and Proteomics 1784:1873–1898.

39. Kranz RG, Richard-Fogal C, Taylor J-S, Frawley ER. 2009. Cytochrome *c* Biogenesis: Mechanisms for Covalent Modifications and Trafficking of Heme and for Heme-Iron Redox Control. Microbiology and Molecular Biology Reviews 73:510–528.

40. Ferousi C, Lindhoud S, Baymann F, Hester ER, Reimann J, Kartal B. 2019. Discovery of a functional, contracted heme-binding motif within a multiheme cytochrome. Journal of Biological Chemistry 294:16953–16965.

41. Hartshorne RS, Kern M, Meyer B, Clarke TA, Karas M, Richardson DJ, Simon J. 2007. A dedicated haem lyase is required for the maturation of a novel bacterial cytochrome *c* with unconventional covalent haem binding. Mol Microbiol 64:1049–1060.

42. Edwards MJ, Richardson DJ, Paquete CM, Clarke TA. 2020. Role of multiheme cytochromes involved in extracellular anaerobic respiration in bacteria. Protein Science 29:830–842.

43. Hallgren J, Tsirigos KD, Pedersen MD, Almagro Armenteros JJ, Marcatili P, Nielsen H, Krogh A, Winther O. 2022. DeepTMHMM predicts alpha and beta transmembrane proteins using deep neural networks https://doi.org/10.1101/2022.04.08.487609.

44. Zeth K, Thein M. 2010. Porins in prokaryotes and eukaryotes: common themes and variations. Biochemical Journal 431:13–22.

45. Steinegger M, Söding J. 2017. MMseqs2 enables sensitive protein sequence searching for the analysis of massive data sets. Nat Biotechnol 35:1026–1028.

46. Diao M, Dyksma S, Koeksoy E, Ngugi DK, Anantharaman K, Loy A, Pester M. 2023. Global diversity and inferred ecophysiology of microorganisms with the potential for dissimilatory sulfate/sulfite reduction. FEMS Microbiol Rev 47.

47. Yan Z, Wang M, Ferry JG. 2017. A ferredoxin- and f420h2-dependent, electron-bifurcating, heterodisulfide reductase with homologs in the domains Bacteria and Archaea. mBio 8.

48. Berrisford JM, Baradaran R, Sazanov LA. 2016. Structure of bacterial respiratory complex I. Biochimica et Biophysica Acta (BBA) - Bioenergetics 1857:892–901.

49. Kuhns M, Trifunović D, Huber H, Müller V. 2020. The Rnf complex is a Na+ coupled respiratory enzyme in a fermenting bacterium, Thermotoga maritima. Commun Biol 3:431.

50. Jochum LM, Schreiber L, Marshall IPG, Jørgensen BB, Schramm A, Kjeldsen KU. 2018. Single-Cell Genomics Reveals a Diverse Metabolic Potential of Uncultivated Desulfatiglans-Related Deltaproteobacteria Widely Distributed in Marine Sediment. Front Microbiol 9.

51. Wolfe AJ. 2005. The Acetate Switch. Microbiology and Molecular Biology Reviews 69:12–50.

52. Yang S, Lv Y, Liu X, Wang Y, Fan Q, Yang Z, Boon N, Wang F, Xiao X, Zhang Y. 2020. Genomic and enzymatic evidence of acetogenesis by anaerobic methanotrophic archaea. Nat Commun 11:3941.

53. Didonato RJ, Young ND, Butler JE, Chin KJ, Hixson KK, Mouser P, Lipton MS, Deboy R, Methé BA. 2010. Genome sequence of the deltaproteobacterial strain naphs2 and analysis of differential gene expression during anaerobic growth on naphthalene. PLoS One 5:e14072.

54. Xie X, Spiteller D, Huhn T, Schink B, Müller N. 2020. Desulfatiglans anilini Initiates Degradation of Aniline With the Production of Phenylphosphoamidate and 4-Aminobenzoate as Intermediates Through Synthases and Carboxylases From Different Gene Clusters. Front Microbiol 11.
