# Supplementary material for: Desulfatiglans-related bacteria associated with conductive mineral particles in marine subsurface sediments
Source: mBio. 2026 Jun 15;17(7):e00838-26. doi: 10.1128/mbio.00838-26 (PMC13343851; doi:10.1128/mbio.00838-26)
Supplement: Supplemental material — Supplemental tables and figures. [file mbio.00838-26-s0002.docx]

**Supplementary Information Tables**

**Table S1:** Quality of *Desulfatiglans*-related metagenome assembled genome (MAG) bins. All MAGs classify within the order *Desulfatiglandales* except SED112_bin74. Single cell amplified genomes (SAG) were taken from Jochum et al. (2018) (1). Clade A and B refer to inferred non-sulfate reducing and sulfate-reducing phylogenetic lineages. (*) 16S rRNA gene sequence related to *Desulfatiglans*, genome related to SEEP-SRB1 (see Fig. S6). (#) MAG does not include a 16S rRNA gene. See Fig. S9 for detailed taxonomic classification.

| MAG bin | Size (Mbp) | Completeness (%) | Contami-nation (%) | Clade | GTDB classification  Family |
| --- | --- | --- | --- | --- | --- |
| SED112_bin125 | 5.6 | 91 | 11 | A | B25-G16 |
| SED114_bin110 | 5.2 | 91 | 7.9 | A | B25-G16 |
| SED134_bin25 | 4.2 | 7.4 | 3.9 | A | B25-G16 |
| SED112_bin92 | 3.1 | 77 | 6.4 | A | B25-G16 |
| SED114_bin113 | 3.7 | 89 | 8.8 | A | B25-G16 |
| SED134_bin21 | 4.8 | 83 | 16 | A | B25-G16 |
| SED112_bin71 | 2.4 | 87 | 1.5 | A | B25-G16 |
| *SED112_bin74 | 4.1 | 65 | 2.9 | A | C00003060 |
| SED114_bin111 | 3.5 | 83 | 11 | A | B25-G16 |
| SED134_bin47 | 2.0 | 68 | 3.3 | A | B25-G16 |
| SED114_bin88 | 4.2 | 91 | 6.2 | A | B25-G16 |
| SED134_bin123 | 4.7 | 91 | 6.5 | A | B25-G16 |
| SED112_bin79^#^ | 15 | 61 | 34 | A | B25-G16 |
| SED114_bin174^#^ | 1.8 | 54 | 3.7 | A | B25-G16 |
| SED134_bin179^#^ | 1.9 | 70 | 5.5 | A | B25-G16 |
| SED134_bin83^#^ | 3.6 | 57 | 4.1 | A | B25-G16 |
| SED112_bin84 | 3.9 | 89 | 2.0 | B | Desulfatiglandaceae |
| SED114_bin167 | 19 | 63 | 30 | B | Desulfatiglandaceae |
| SED114_bin152 | 7.1 | 92 | 26 | B | Desulfatiglandaceae |
| SED134_bin178 | 4.3 | 76 | 16 | B | Desulfatiglandaceae |
| SED114_bin159 | 5.5 | 93 | 17 | B | Desulfatiglandaceae |
| SED134_bin161 | 4.3 | 93 | 4.5 | B | Desulfatiglandaceae |
| SED112_bin93 | 3.0 | 78 | 4.3 | B | Desulfatiglandaceae |
| SED112_bin108^#^ | 1.6 | 56 | 1.6 | B | Desulfatiglandaceae |
| SED114_bin177^#^ | 3.2 | 71 | 6.6 | B | Desulfatiglandaceae |
| SED114_bin157^#^ | 1.8 | 35 | 1.8 | B | Desulfatiglandaceae |
| SED114_bin47^#^ | 2.3 | 69 | 1.1 | B | Desulfatiglandaceae |
| SED134_bin128^#^ | 4.5 | 49 | 10.3 | B | B25-G16 |
| SED134_bin111^#^ | 4.5 | 90 | 4.0 | B | Desulfatiglandaceae |
| SAG14 | 0.9 | 50 | 0.6 | A | B25-G16/JADFWK01 |
| SAG13 | 1.5 | 25 | 1.7 | B | Desulfatiglandaceae |
| SAG3 | 2.5 | 55 | 1.6 | B | HGW-15 |
| SAG5 | 0.8 | 19 | 0 | B | Desulfatiglandaceae |
| SAG8 | 1.4 | 31 | 0.3 | B | Desulfatiglandaceae |

**Table S2.** Average Nucleotide Identity (ANI) values among clade A MAGs. The first row lists MAG names and their sizes. Values in parentheses indicate the alignment fraction used in the ANI calculation, defined as the number of 3,000 nt fragments from the query MAG (first column) that aligned to the reference MAG, divided by the total number of 3,000 nt fragments in the query MAG. ANI values equal to or greater than the species boundary threshold of 95% (2) are highlighted in red. bd: below detection threshold of 75% pairwise ANI.


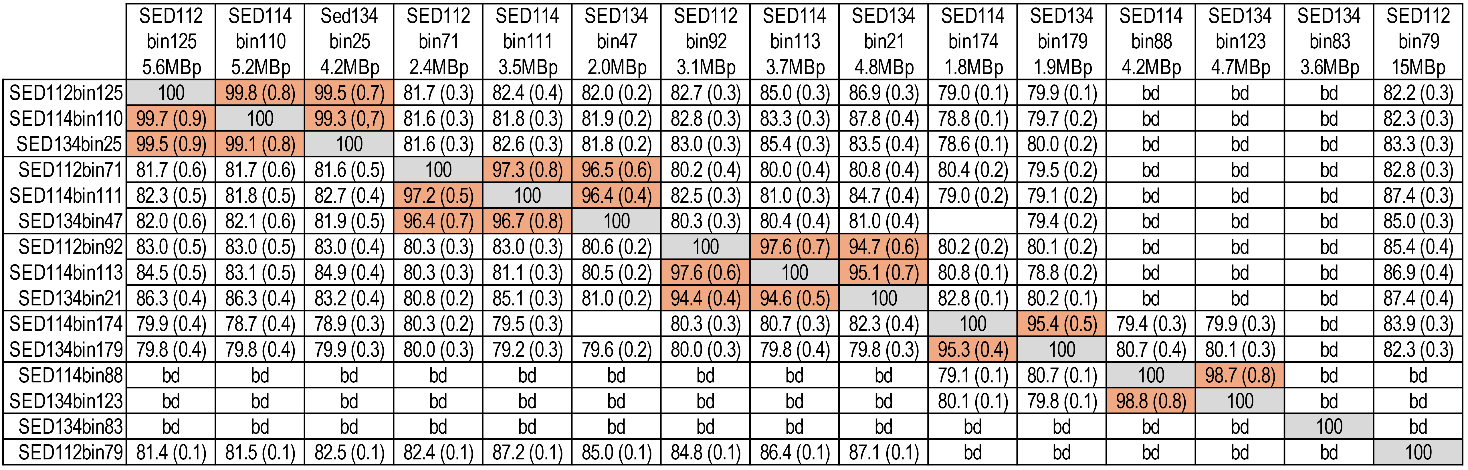


**Supplementary Information Figures**


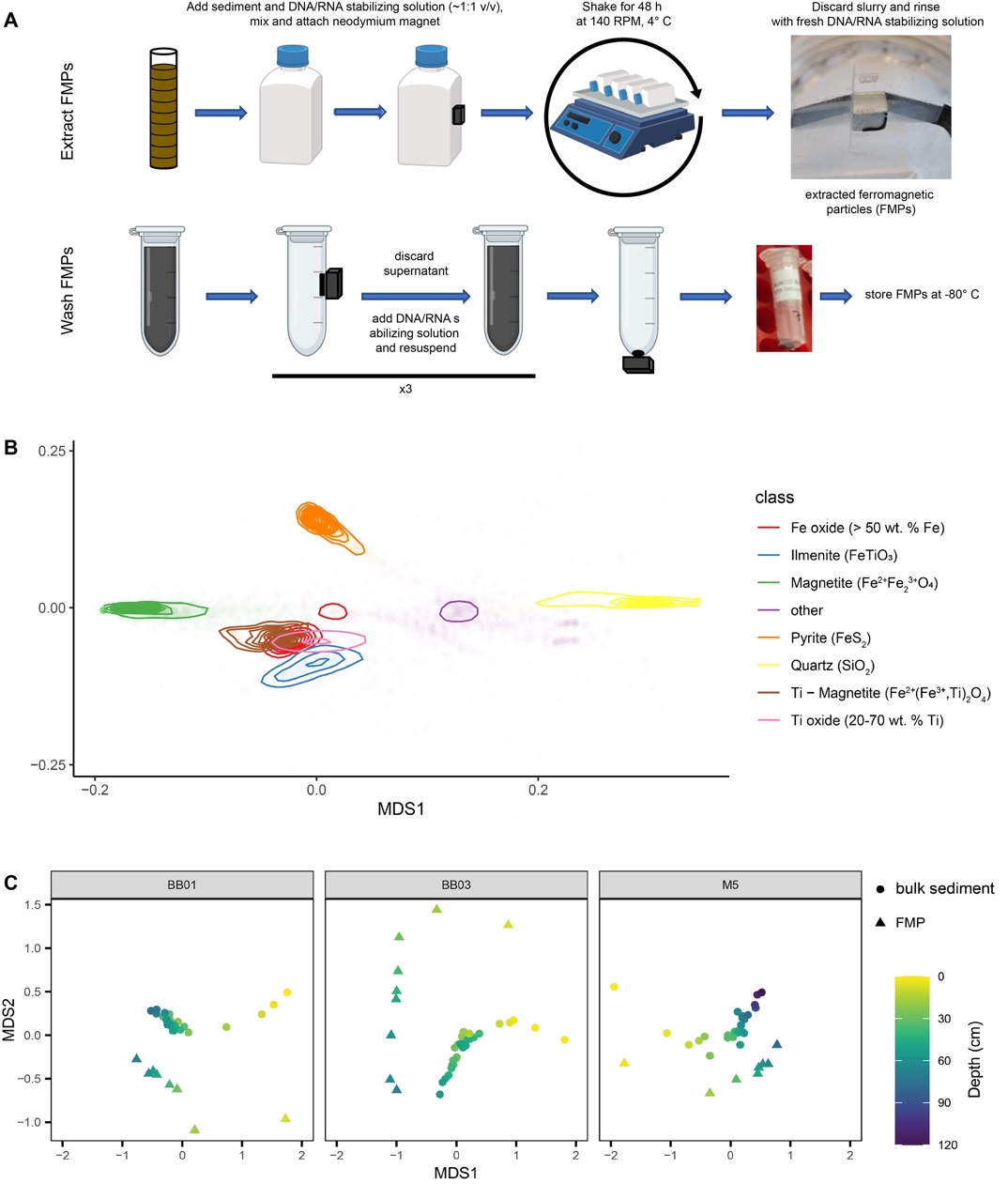


**Figure S1**: Procedure to extract ferromagnetic particles (FMPs) from bulk sediments. Sediment intervals were transferred to sterile plastic bottles filled with an equal amount of DNA and RNA stabilizing solution. After 48 h of gently shaking, extracted FMPs were transferred to micro centrifuge tubes, washed and stored at -80° C.


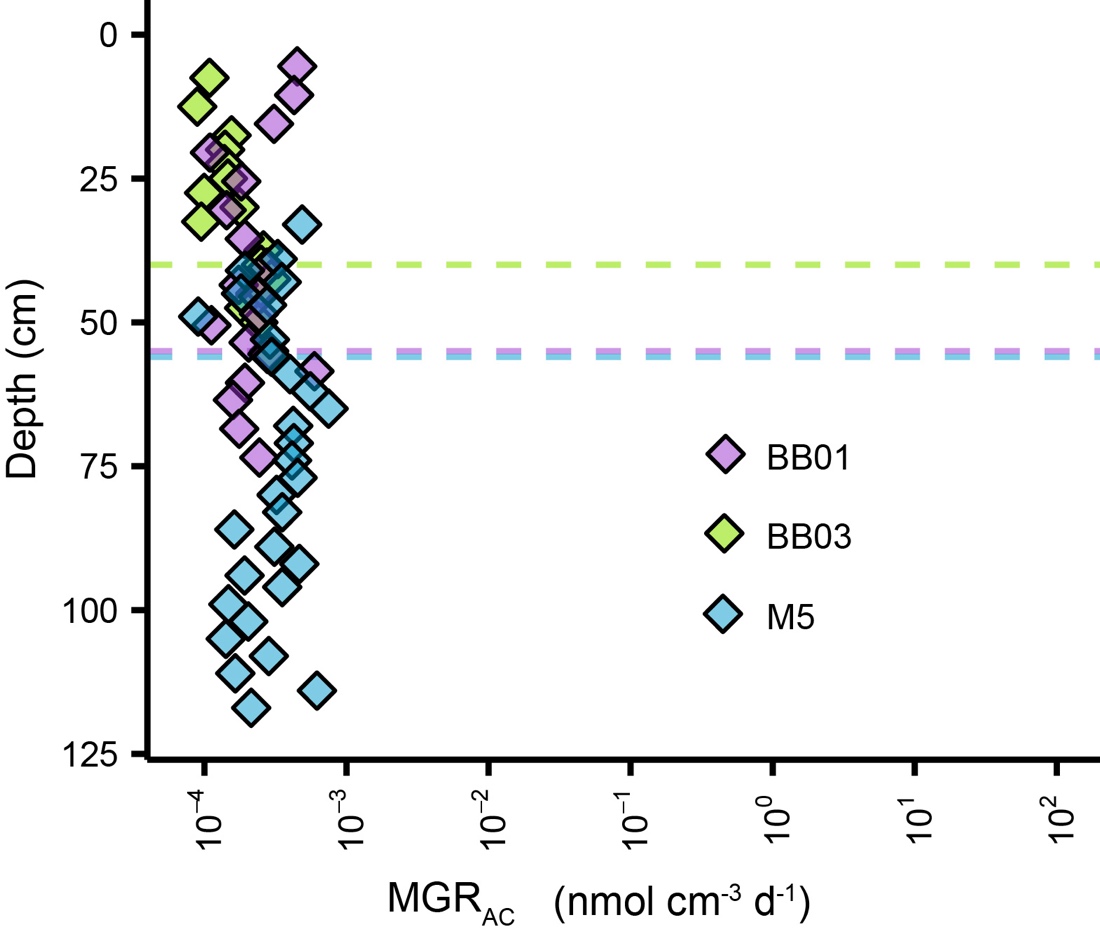


**Figure S2**: Sediment depth distribution of rates of acetoclastic methanogenesis (MGR_AC_). Dashed lines indicate the depth of the SMT of the respective station. Data from station M5 were taken from Beulig et al. (2019) (3) and vertically aligned based on porewater sulfate and methane concentration profiles.


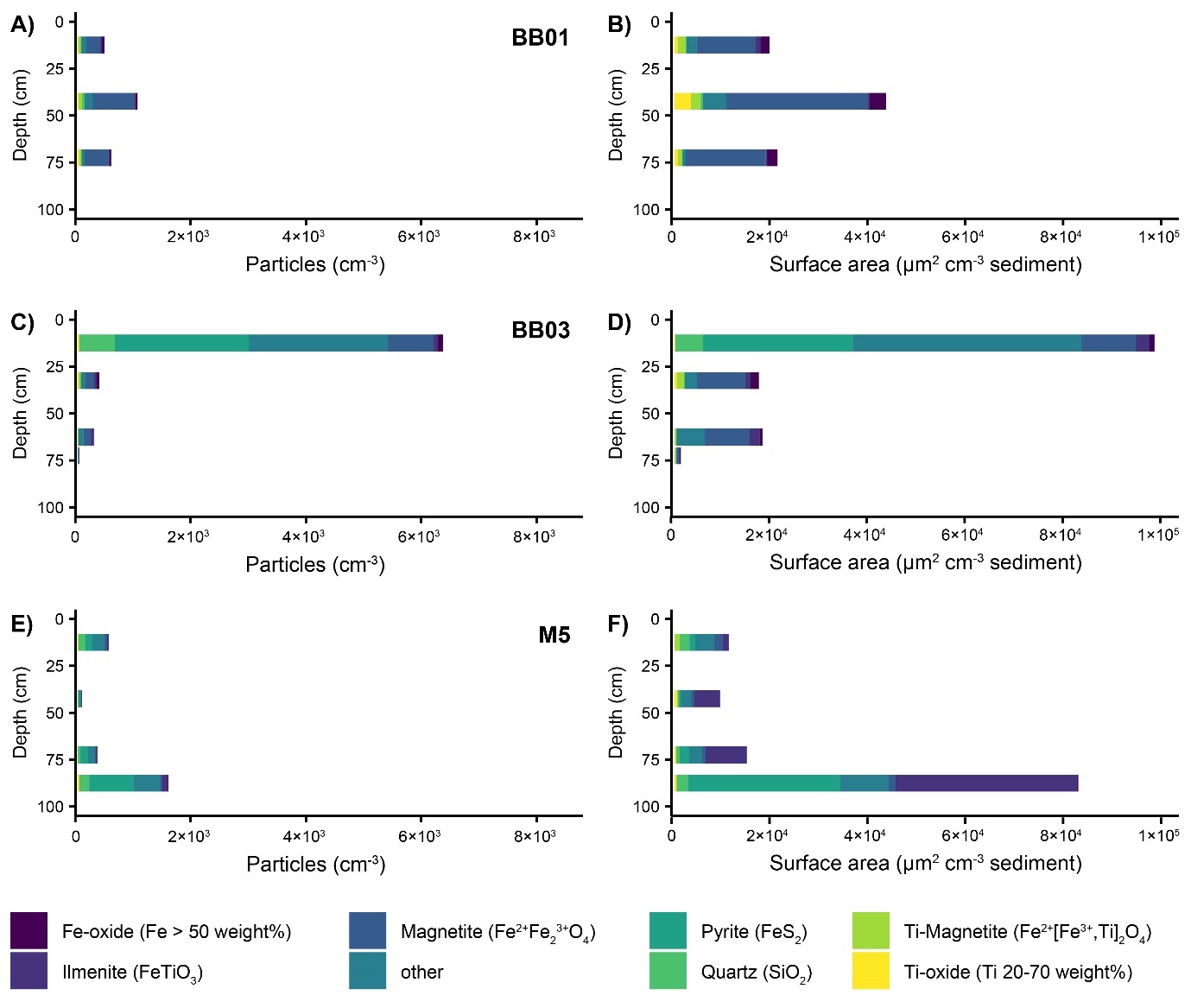


**Figure S3**: Abundance and estimated surface area of extracted ferromagnetic particles from sediment core samples from Bornholm Basin station BB01 (**A** & **B**), BB03 (**C** & **D**) and Aarhus Bay station M5 (**E** & **F**).


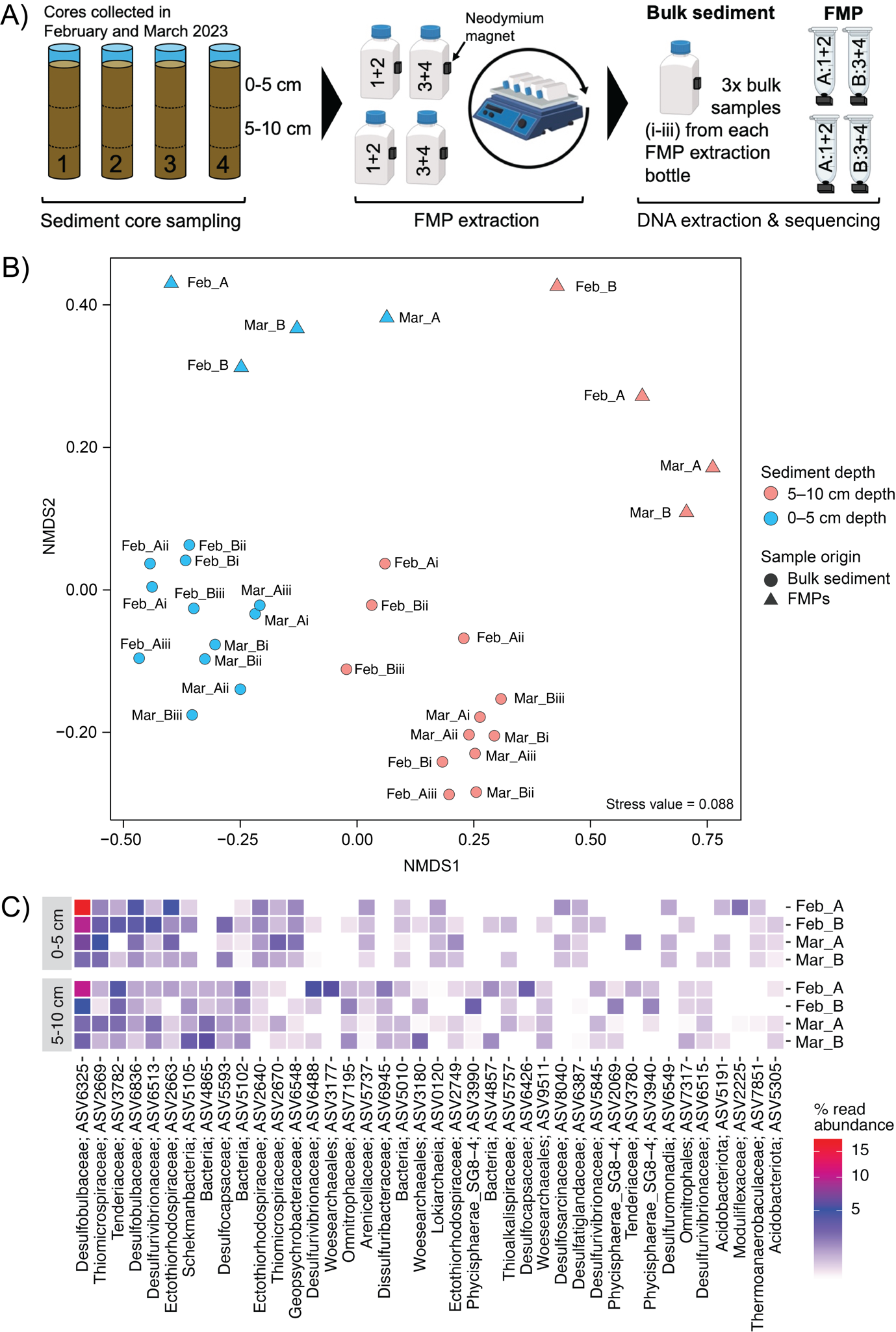


**Figure S4**: Validation of the reproducibility of 16S rRNA gene amplicon-based profiling of microbial communities associated with ferro-magneticle particles (FMPs) extracted from marine sediment samples.

(**A**) Conceptual outline of the experimental design. Four 15 cm long and 5 cm diameter sediment cores were collected in Aarhus Harbor (latitude 56.167581, longitude 10.223893) in February and in March 2023. The 0-5 and 5-10 cm sections of replicate cores were respectively pooled and slurried in DNA and RNA stabilizing salt solution for magnet-based extraction of ferromagnetic particles (FMPs). Hereby four slurries were obtained for each depth interval - two (A and B) from each sampling month. DNA was extracted from 3 subsamples (i-iii) from each slurry and from each of the eight FMP extracts. Finally, the microbial community of the samples was determined by 16S rRNA gene PCR amplicon sequencing. All methods were described in the main text of the manuscript.

(**B**) Non-Metric Multidimensional Scaling-based ordination analysis of the ASV-level composition of FMP-associated and bulk sediment microbial communities. The ordination was based on a Bray-Curtis dissimilarity matrix.

(**C**) Heatmap showing the identity and taxonomic affiliation of the top-most 40 abundant ASVs uniquely present in FMP-associated communities as compared to the bulk sediment communities. Depending on the sample, between 14 and 29% of the ASVs observed in FMP sequence libraries were absent in the bulk sediment libraries. These uniquely FMP-associated ASV constituted 5-17% of the reads in the FMP sequence libraries. The scale bar shows the percent relative read abundance of ASVs among the uniquely FMP-associated sequence reads of a given library. Note that the abundant ASVs uniquely associated with FMPs appear non-randomly assembled as these same ASVs are consistently observed across independent replicates.


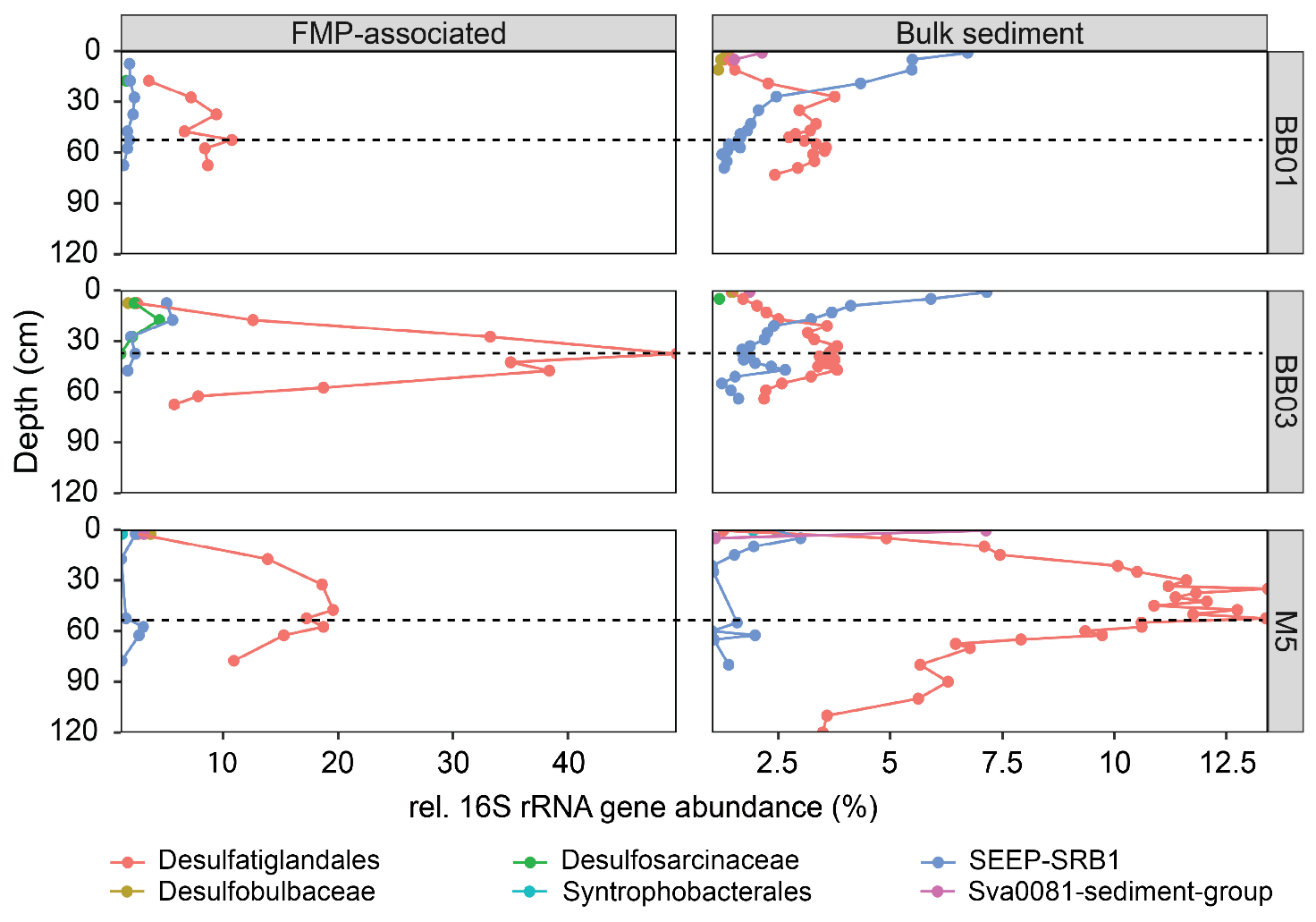


**Figure S5:** Identity and sediment depth distribution of different taxonomic lineages of the phyla *Desulfobacterota* based on 16S rRNA gene amplicon sequencing of ferromagnetic particles (FMP) and from bulk sediment samples from sampling stations BB01 and BB03 in the Bornholm Basin and station M5 in Aarhus Bay. Only lineages with a relative abundance exceeding 1% were included in the figure. Dashed lines indicate the depth of the sulfate-methane transition (SMT).


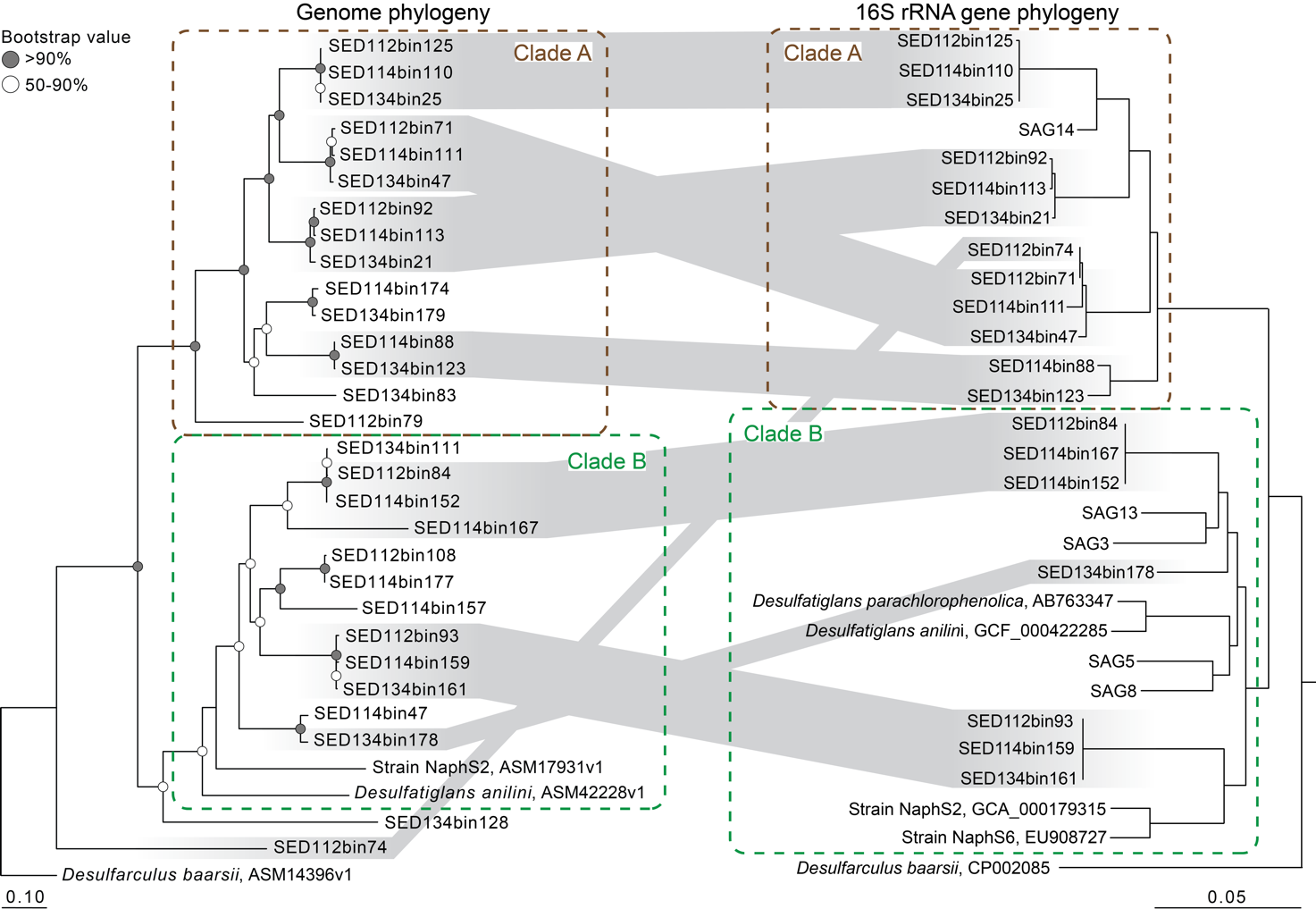


**Figure S6:** Phylogeny of metagenome assembled genomes (MAGs [name beginning with “SED”]) and single cell amplified genomes (SAGs) affiliated with members of the order *Desulfatiglandales*. Clade A represents taxa lacking the genetic potential for dissimilatory sulfate reduction, while clade B represents predicted (MAGs/SAGs) and known (characterized isolates) dissimilatory sulfate reducers. (**A**) Genome-resolved phylogeny based on a concatenated alignment of inferred protein sequences of 120 bacterial marker genes as identified by the “gtdbtk identify” command of the GTDB-Tk software toolkit (4). Depending on the coverage the MAGs/genomes missed between 0 and 56 of the 120 marker genes (average±st.dev = 25±19). The phylogenetic tree was estimated by maximum likelihood analysis using IQ-TREE v. 1.6.12 (5) with 100 bootstrap replicates. A ModelFinder (6) run as implemented in IQ-TREE identified LG+F+R4 as the best model for the analysis. (**B**). 16S rRNA gene sequence-based phylogeny. The phylogenetic tree was estimated by distance matrix analysis using the pos_var_ssuref:bacteria filter of the Silva SSU Ref v138 ARB database (7) for selecting alignment positions for the analysis. Scale bars show 10 and 5% estimated sequence divergence, respectively. See Fig. S9 for a detailed taxonomic classification of MAGs and SAGs.
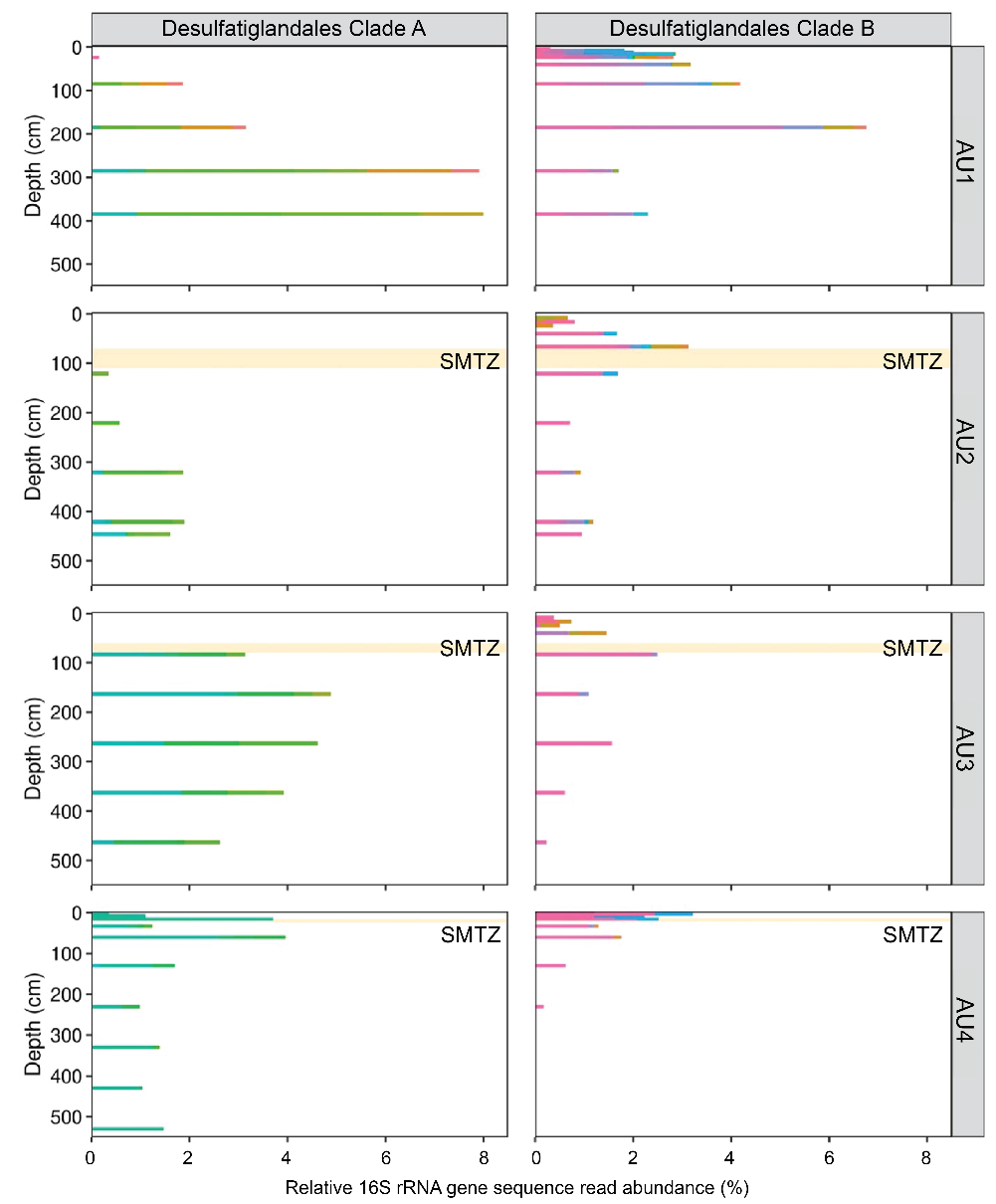


**Figure S7:** Sediment-depth distribution of *Desulfatiglandales*-clade A and B affiliated 16S rRNA gene sequence ASVs at 4 different marine sampling sites located around Denmark. The datasets and geochemical data were taken from Deng et al. (2020) (8) and Marshall et al. (2019) (9). Station reference to the right (AU1-4 and SKA1-4) refers to the naming by Deng et al. 2020 and Marshall et al. 2019, respectively. Orange rectangle indicates the Sulfate-Methane-Transition zone. In (**A**) methane is undetectable and sulfate expands across all sampled depths (mM concentrations). Filling colors indicate relative 16S rRNA gene abundances on ASV level.


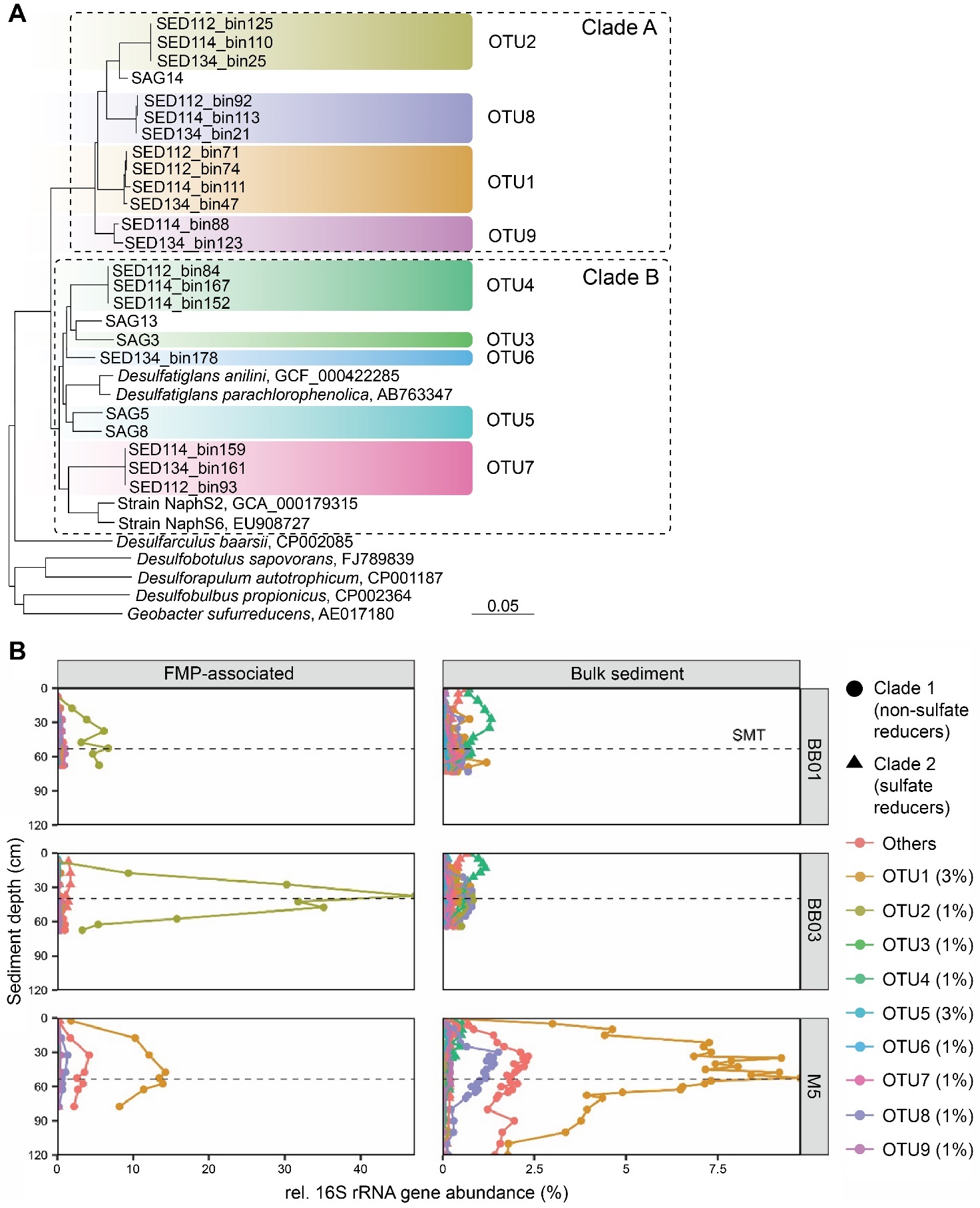


**Figure S8**: (**A**) 16S rRNA gene sequence-based phylogeny of metagenome assembled genomes (MAGs [name beginning with “SED”]) and single cell amplified genomes (SAGs) affiliated with members of the genus *Desulfatiglans*. Clade A represents taxa lacking the genetic potential for dissimilatory sulfate reduction, while clade B represents predicted (MAGs/SAGs) and known (characterized isolates) dissimilatory sulfate reducers. The tree was estimated by distance matrix analysis using the pos_var_ssuref:bacteria filter of the Silva SSU Ref v138 ARB database (7) for selecting alignment positions for the analysis. The scalebar shows 5% estimate sequence divergence. The MAGs were derived from metagenomic sequencing of sediment samples from station BB03 in the Bornholm Basin while SAGs were derived from a previous study of sediment samples from Aarhus Bay station M5 (1). *Desulfatiglans*-affiliated sequence reads from 16S rRNA gene amplicon sequence libraries from ferromagnetic particle (FMP) and sediment samples from station BB01 (Bornholm Basin), BB03 and M5 (see Fig. S5) were mapped onto the MAG and SAG sequences by clustered into operational taxonomic units (OTUs) with either a 1% or a 3% sequence dissimilarity cutoff. The OTU clustering was done using Mothur with the Average Neighbor method (10). Nine different OTUs were identified which included both amplicon library reads and MAG/SAG sequences. (**B**) Sediment depth distribution of OTUs at the three different sampling stations. Amplicon library sequences not forming an OTU with a MAG or a SAG sequence were grouped in the “Others” category. The depth of the sulfate-methane transition (SMT) zone is indicated by dotted lines.


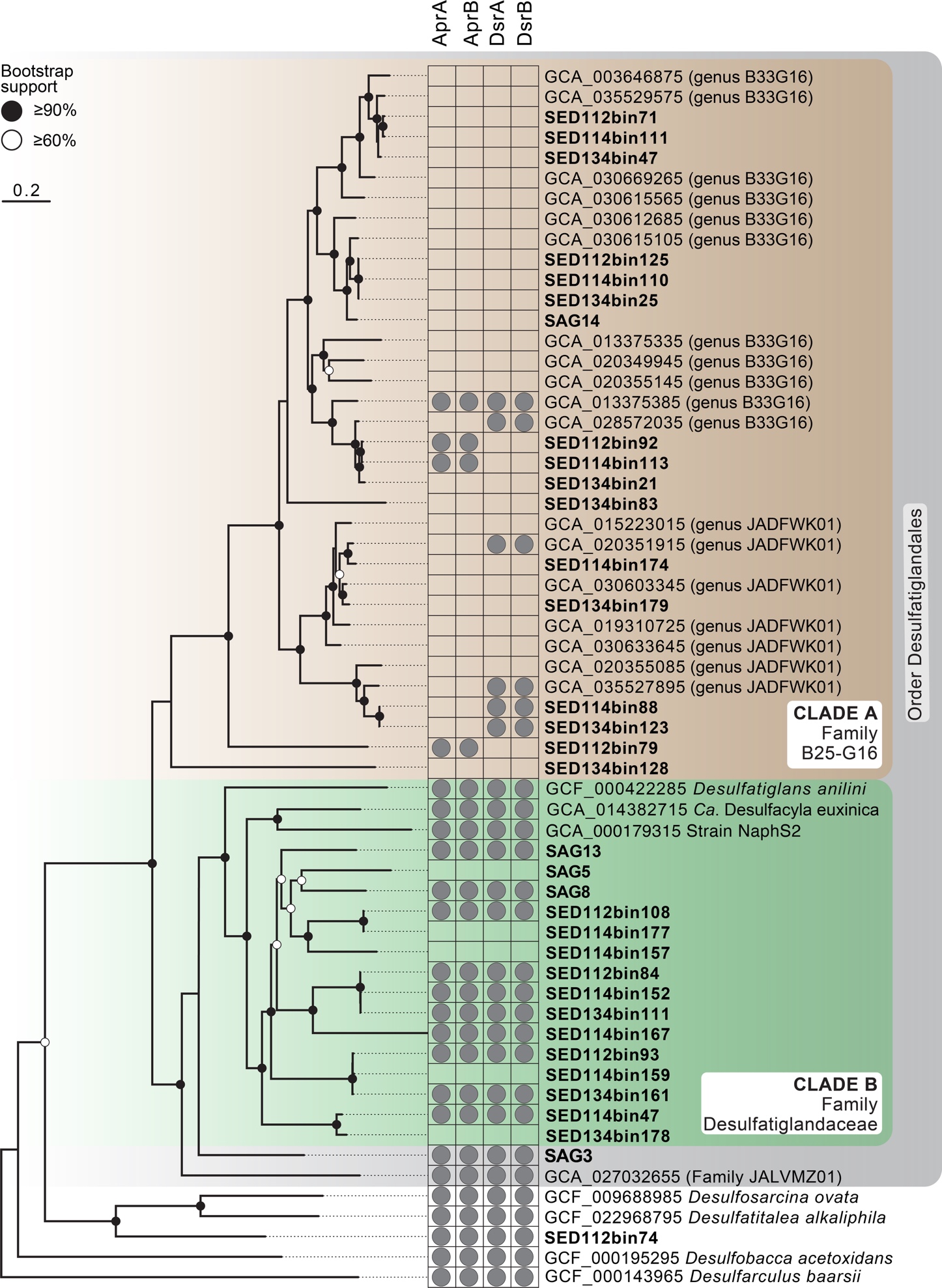


**Figure S9:** Genome-resolved phylogeny, taxonomic classification and genetic potential for dissimilatory sulfate reduction of metagenome assembled genomes (MAGs) and single cell amplified genomes (SAGs) affiliated with members of the genus *Desulfatiglans* and classified within the order *Desulfatiglandales* by the “gtdbtk classify” command of the GTDB-Tk software toolkit (4) version 2.5.2, database release 226. Clade A represents taxa lacking the genetic potential for dissimilatory sulfate reduction, while clade B represents predicted (MAGs/SAGs) and known (characterized isolates) dissimilatory sulfate reducers. Clade A and B members classify within the GTDB families B25-G16 and *Desulfatiglandaceae*, respectively. The phylogenetic tree was estimated by maximum likelihood analysis using IQ-TREE v. 1.6.12 (5) with 100 bootstrap replicates. A ModelFinder (6) run as implemented in IQ-TREE identified LG+F+R5 as the best model for the analysis. Genomes encoding the dissimilatory sulfate-reduction marker genes AprAB (adenosine 5′-phosphosulfate reductase subunit A and B) and DsrAB (dissimilatory sulfite reductase subunit A and B) are indicated by grey circles. Scale bar show 10 % estimated sequence divergence.


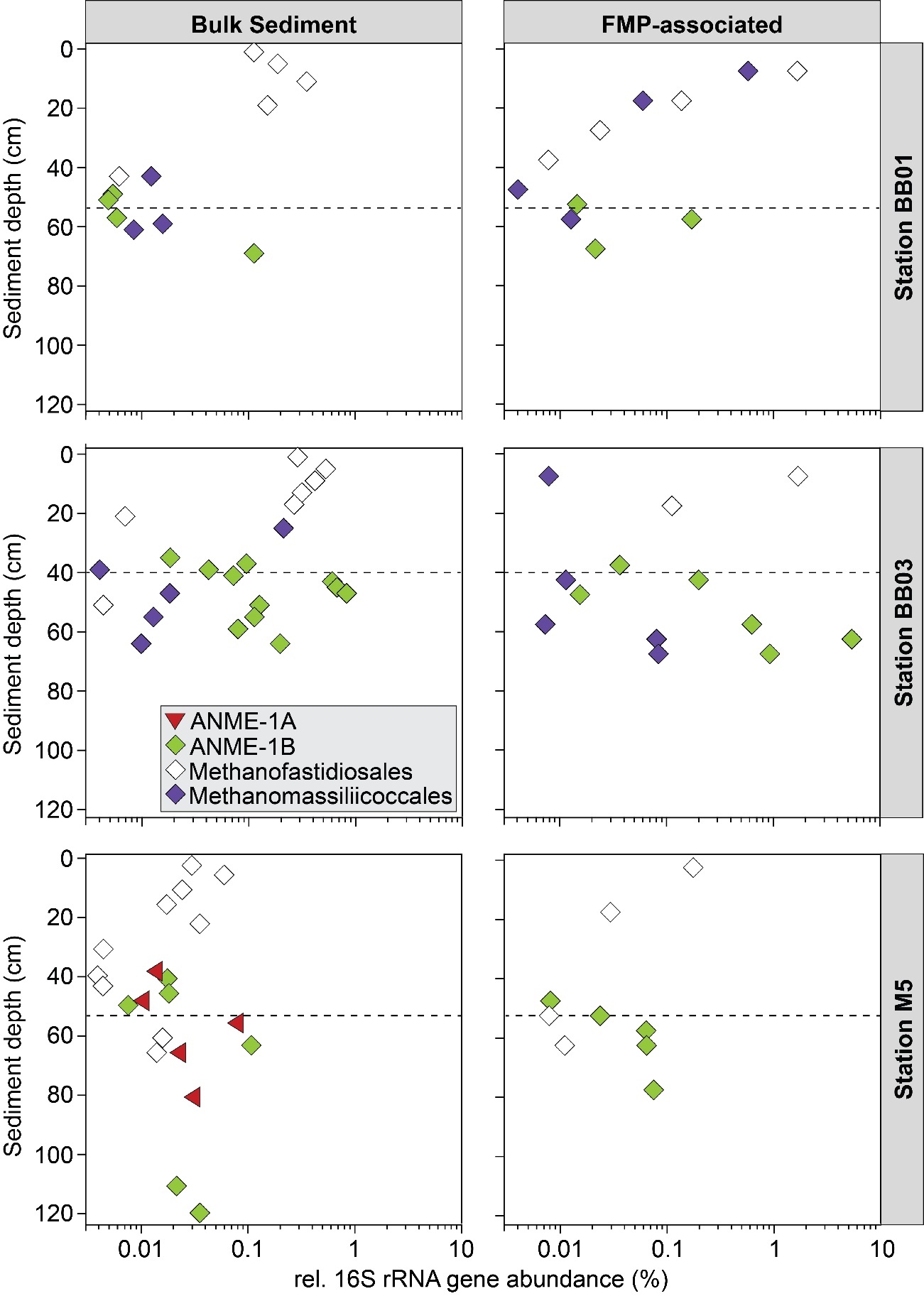


**Figure S10**: Sediment depth distribution of anaerobic methane-cycling archaea at sampling stations BB01 and BB03 in the Bornholm Basin and station M5 in Aarhus Bay based on 16S rRNA gene amplicon sequence libraries generated from universal primers from ferromagnetic particle (FMP) or bulk sediment samples. The depth of the sulfate-methane transition zone in the sediments is indicated by dashed lines.


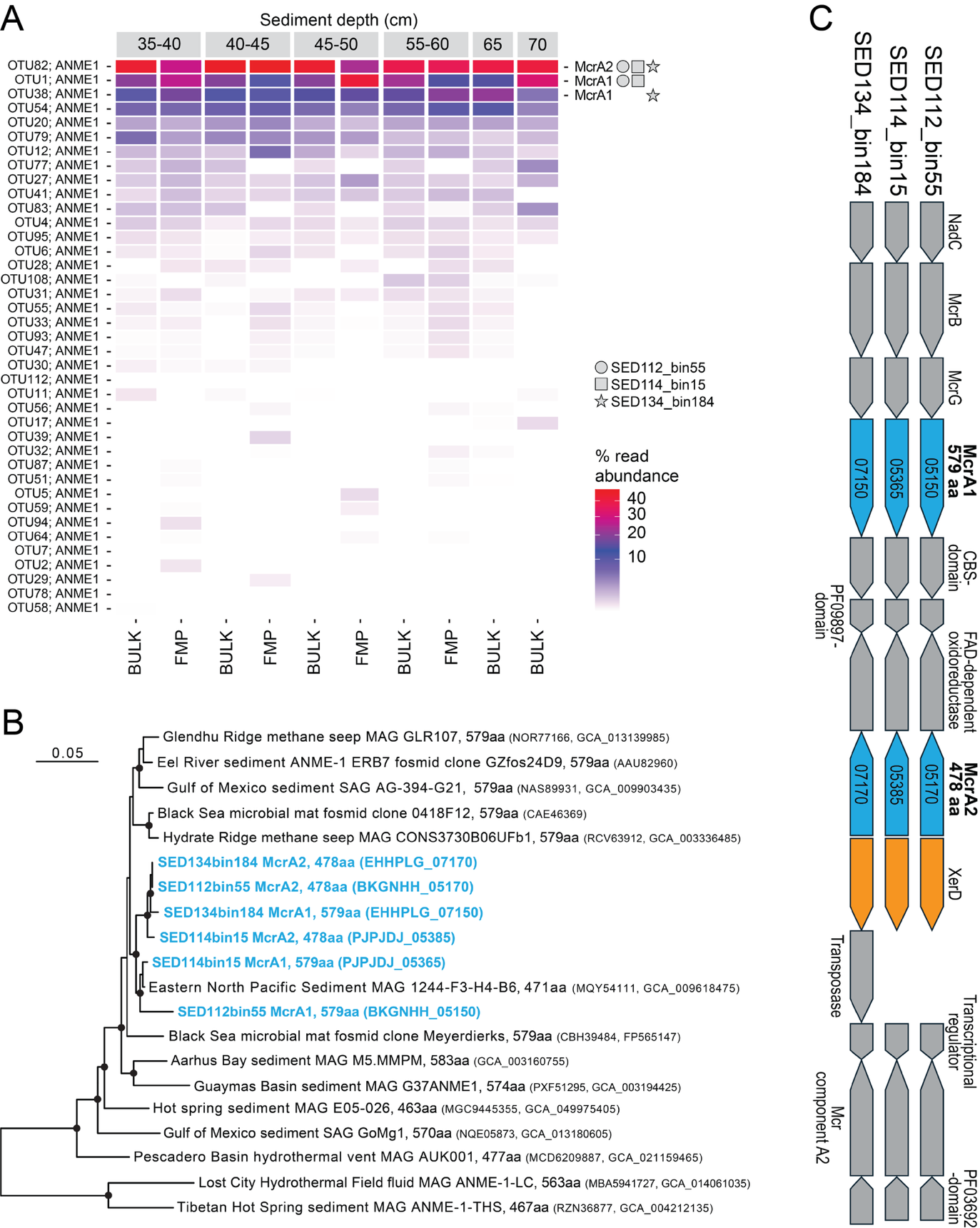


**Figure S11**. (A) Sediment-depth distribution and phylogentic affiliation of methyl coenzyme M reductase subunit A (McrA) protein sequence OTUs derived from *mcrA* gene amplicon sequence libraries from ferromagnetic particles (FMPs) and bulk sediment (BULK) samples from station sampling station BB03. Observed OTUs were consistently affiliated with ANME1 archaea. The three most abundant McrA OTUs were identical to McrA proteins encoded in three ANME1-affiliared MAGs (Sed112bin55, Sed114bin15 and Sed134bin183) obtained from metagenomic sequencing of the same sampling station. The phylogeny of these McrA protein sequences is shown in panel (B). The shown tree was estimated by RaxML (version 7.2.8, (11)) analysis using the WAG substitution matrix. Nodes receiving ≥70% bootstrap support (n=100 replicates) are labelled with solid circles. The scale bar shows 5% estimated sequence divergence. (C) Gene neighborhood of the two *mcrA* gene copies present in the Sed112bin55, Sed114bin15 and Sed134bin183 ANME1-affiliated MAGs. The second *mcrA* gene (labeled McrA2) copy is located next to a XerD-type recombinase-encoding gene and its gene product is truncated in its N-terminal part by 100 amino acids relative to the product of the other *mcrA* gene (labeled McrA1). See SI Data and Figure S12 for details.


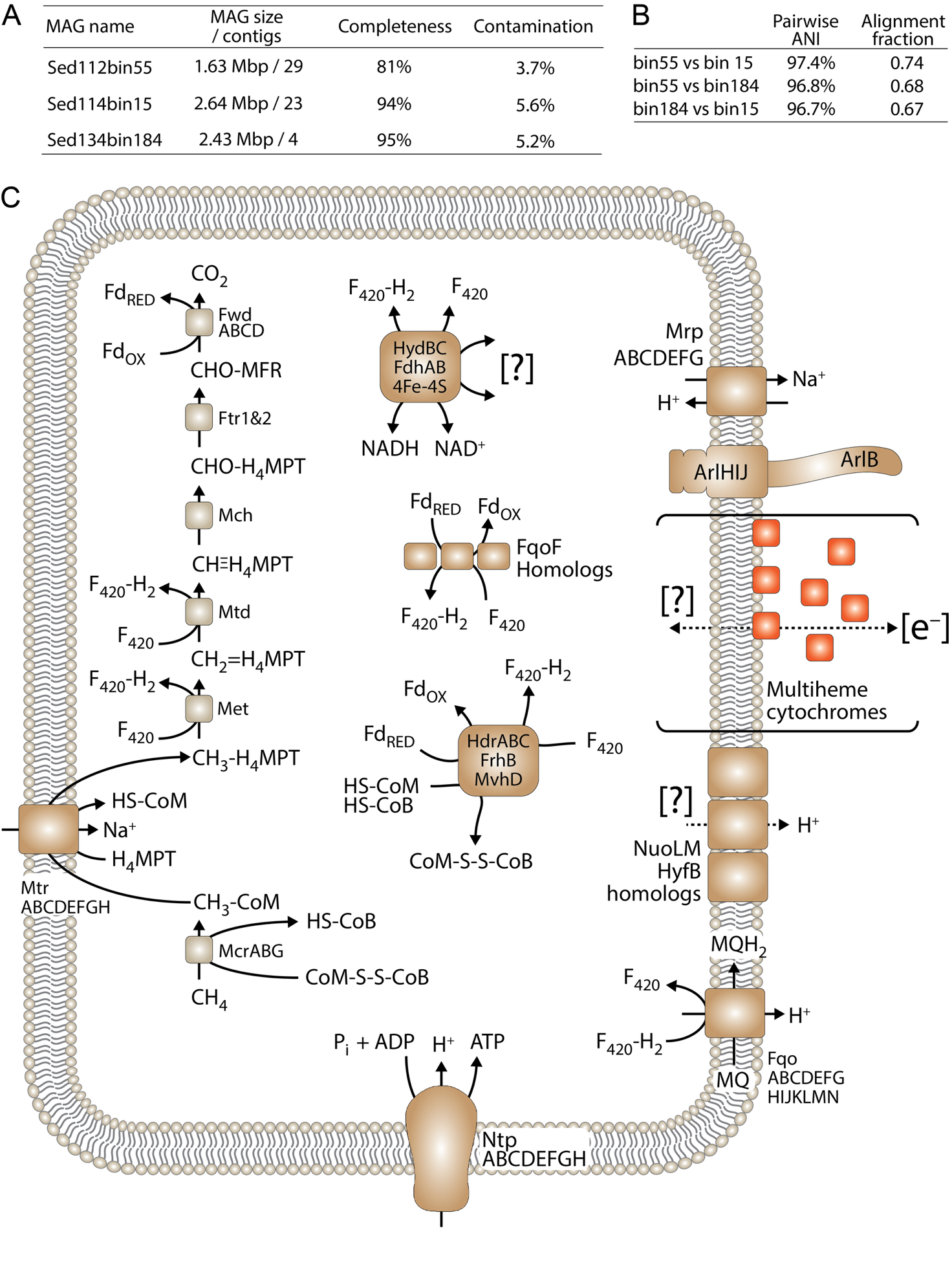


**Figure S12**. MAG statistics (**A and B**) and MAG-inferred metabolic model (C) of ANME1 archaea predominant in the sediment of sampling station BB03 and associated with ferromagnetic particles. See Table S2 for details on ANI and alignment fraction. Mcr, Methyl coenzyme M reductase. Mtr, Tetrahydromethanopterin S-methyltransferase. Met, 5,10-methylenetetrahydrofolate reductase. Mtd, Coenzyme F420-dependent N(5),N(10)-methenyltetrahydromethanopterin dehydrogenase. Mch, Methenyltetrahydromethanopterin cyclohydrolase. Ftr, Formylmethanofuran-tetrahydromethanopterin formyltransferase. Fwd, Formylmethanofuran dehydrogenase. Mrp, Multisubunit Na+/H+ antiporter. Ntp, V-type ATP synthase. Fqo, Coenzyme F420:quinone oxidoreductase. Arl, archaellum. Dotted lines and questions marks indicate hypothetical processes. See SI Data for details.

References (in this document)

1. Jochum LM, Schreiber L, Marshall IPG, Jørgensen BB, Schramm A, Kjeldsen KU. 2018. Single-cell genomics reveals a diverse metabolic potential of uncultivated Desulfatiglans-related deltaproteobacteria widely distributed in marine sediment. Front Microbiol 9:1–16.

2. Richter M, Rosselló-Móra R. 2009. Shifting the genomic gold standard for the prokaryotic species definition. Proceedings of the National Academy of Sciences 106:19126–19131.

3. Beulig F, Røy H, McGlynn SE, Jørgensen BB. 2019. Cryptic CH 4 cycling in the sulfate–methane transition of marine sediments apparently mediated by ANME-1 archaea. ISME Journal 13:250–262.

4. Chaumeil PA, Mussig AJ, Hugenholtz P, Parks DH. 2020. GTDB-Tk: A toolkit to classify genomes with the genome taxonomy database. Bioinformatics 36:1925–1927.

5. Nguyen L-T, Schmidt HA, von Haeseler A, Minh BQ. 2015. IQ-TREE: A Fast and Effective Stochastic Algorithm for Estimating Maximum-Likelihood Phylogenies. Mol Biol Evol 32:268–274.

6. Kalyaanamoorthy S, Minh BQ, Wong TKF, von Haeseler A, Jermiin LS. 2017. ModelFinder: fast model selection for accurate phylogenetic estimates. Nat Methods 14:587–589.

7. Quast C, Pruesse E, Yilmaz P, Gerken J, Schweer T, Yarza P, Peplies J, Glöckner FO. 2013. The SILVA ribosomal RNA gene database project: Improved data processing and web-based tools. Nucleic Acids Res 41.

8. Deng L, Bölsterli D, Kristensen E, Meile C, Su C-C, Bernasconi SM, Seidenkrantz M-S, Glombitza C, Lagostina L, Han X, Jørgensen BB, Røy H, Lever MA. 2020. Macrofaunal control of microbial community structure in continental margin sediments. Proc Natl Acad Sci U S A 117:15911–15922.

9. Marshall IPG, Ren G, Jaussi M, Lomstein BA, Jørgensen BB, Røy H, Kjeldsen KU. 2019. Environmental filtering determines family-level structure of sulfate-reducing microbial communities in subsurface marine sediments. ISME J 13:1920–1932.

10. Schloss PD, Westcott SL, Ryabin T, Hall JR, Hartmann M, Hollister EB, Lesniewski RA, Oakley BB, Parks DH, Robinson CJ, Sahl JW, Stres B, Thallinger GG, Van Horn DJ, Weber CF. 2009. Introducing mothur: Open-Source, Platform-Independent, Community-Supported Software for Describing and Comparing Microbial Communities. Appl Environ Microbiol 75:7537–7541.

11. Stamatakis A. 2014. RAxML version 8: a tool for phylogenetic analysis and post-analysis of large phylogenies. Bioinformatics 30:1312–1313.
